# Integrative analysis of RNA polymerase II and transcriptional dynamics upon MYC activation

**DOI:** 10.1101/gr.226035.117

**Published:** 2017-10

**Authors:** Stefano de Pretis, Theresia R. Kress, Marco J. Morelli, Arianna Sabò, Chiara Locarno, Alessandro Verrecchia, Mirko Doni, Stefano Campaner, Bruno Amati, Mattia Pelizzola

**Affiliations:** 1 Center for Genomic Science of IIT@SEMM, Fondazione Istituto Italiano di Tecnologia (IIT), 20139 Milan, Italy;; 2 Department of Experimental Oncology, European Institute of Oncology (IEO), 20139 Milan, Italy

## Abstract

Overexpression of the MYC transcription factor causes its widespread interaction with regulatory elements in the genome but leads to the up- and down-regulation of discrete sets of genes. The molecular determinants of these selective transcriptional responses remain elusive. Here, we present an integrated time-course analysis of transcription and mRNA dynamics following MYC activation in proliferating mouse fibroblasts, based on chromatin immunoprecipitation, metabolic labeling of newly synthesized RNA, extensive sequencing, and mathematical modeling. Transcriptional activation correlated with the highest increases in MYC binding at promoters. Repression followed a reciprocal scenario, with the lowest gains in MYC binding. Altogether, the relative abundance (henceforth, “share”) of MYC at promoters was the strongest predictor of transcriptional responses in diverse cell types, predominating over MYC's association with the corepressor ZBTB17 (also known as MIZ1). MYC activation elicited immediate loading of RNA polymerase II (RNAPII) at activated promoters, followed by increases in pause-release, while repressed promoters showed opposite effects. Gains and losses in RNAPII loading were proportional to the changes in the MYC share, suggesting that repression by MYC may be partly indirect, owing to competition for limiting amounts of RNAPII. Secondary to the changes in RNAPII loading, the dynamics of elongation and pre-mRNA processing were also rapidly altered at MYC regulated genes, leading to the transient accumulation of partially or aberrantly processed mRNAs. Altogether, our results shed light on how overexpressed MYC alters the various phases of the RNAPII cycle and the resulting transcriptional response.

The MYC transcription factor is overexpressed and acts as an oncogenic driver in numerous tumor types. Shedding light on the transcriptional programs driven by MYC is thus a critical area of investigation, with important translational implications. Indeed, numerous studies focused on the analysis of MYC-induced transcriptional responses, typically measuring its binding through ChIP-seq and profiling transcriptional maps by RNA-seq (Kress et al. 2015). A series of papers proposed that, rather than acting as a gene-specific regulator, MYC acts as a general amplifier of transcriptional activity (Lin et al. 2012; Nie et al. 2012). However, our re-analysis of the available data led us to reconsider this model and to conclude that the primary activity of MYC lies in the up- and down-regulation of selected sets of genes, RNA “amplification”—when occurring—being best explained as a secondary consequence (Sabò et al. 2014; Walz et al. 2014; Kress et al. 2015, 2016).

At the mechanistic level, how MYC activates and represses transcription remains to be largely addressed. In particular, a unifying view on the role of MYC in the recruitment and progression of RNA polymerase II (RNAPII) within the transcriptional units of regulated genes and the consequent dynamics of transcriptional regulation is still lacking, owing to a series of reasons. First, the interplay between MYC and RNAPII has been studied based on the simple quantification of the density of RNAPII by ChIP-seq, which is not univocally informative on the various steps of RNAPII loading and progression along the transcriptional units. For example, the change of RNAPII density at promoters is determined by the joint action of recruitment and pause-release dynamics, which cannot be fully resolved based on the ChIP-seq data only (Ehrensberger et al. 2013). Second, the transcriptional responses to MYC activation have been characterized based on the changes in total RNA, neglecting that this is determined by the interplay of three processes: the synthesis of pre-mRNA molecules, the processing of the pre-mRNA into a mature mRNA form, and the degradation of the mature form (Braun and Young 2014). Third, a proper study of the RNAPII and transcriptional dynamics would require a detailed time-course analysis of the transcriptional response to MYC activation, which is currently missing.

In order to overcome these limitations, we set to comprehensively characterize the dynamics of transcriptional regulation following MYC activation in mouse fibroblasts. To this purpose, we profiled nascent and total RNA, alongside MYC and RNAPII binding, and used mathematical modeling to quantify the kinetic rates governing the synthesis, processing, and degradation of mRNA and regulating RNAPII loading and progression through the transcriptional units.

## Results

### Relationship between MYC binding and gene regulation

To address how binding of MYC to promoters affects gene regulation, we exploited a series of ChIP- and RNA-seq data sets with activated MYC and control samples. The first three were derived from in vitro models, including the post-translational activation of a Myc-ER chimera in mouse 3T9^MYC-ER^ fibroblasts (Sabò et al. 2014), as well as the conditional expression of recombinant tet-MYC protein in human osteosarcoma (U2OS^tet-MYC^) (Walz et al. 2014) and B-cell lines (P493-6) (Lin et al. 2012). Two other data sets were based on tumors arising in MYC-transgenic mice, including Eµ-*myc* lymphomas (Sabò et al. 2014) and tet-MYC liver carcinomas (Kress et al. 2016), each confronted with its normal tissue counterpart (Supplemental Table S1).

**Figure 1.**
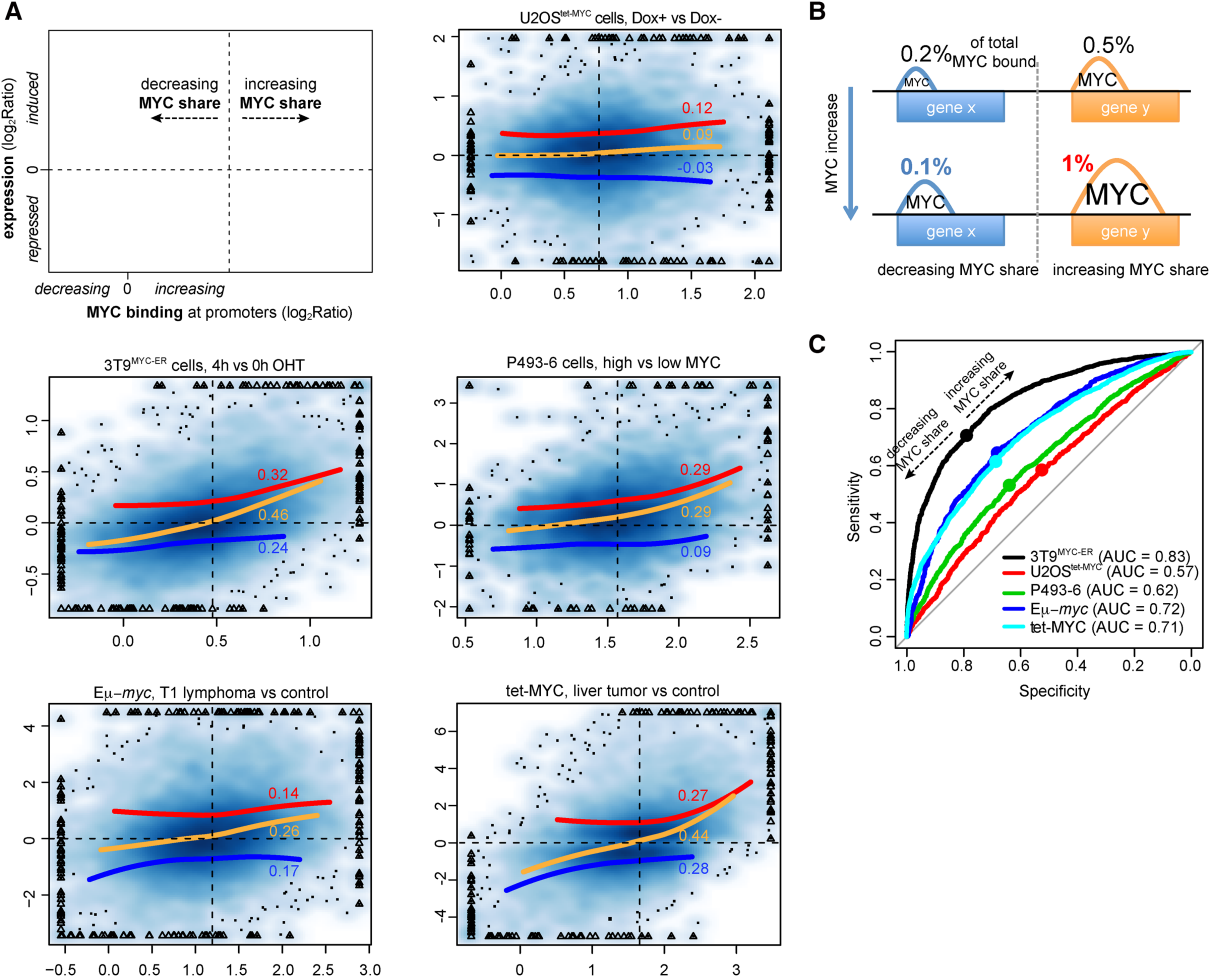
Relationship between MYC binding and gene regulation. (*A*) Density scatter plots (darker colors for higher density) of the variation of MYC ChIP-seq signal within promoters (*x*-axis) vs, the corresponding transcriptional variation (total RNA-seq, *y*-axis). The red, blue, and orange lines capture the trend for induced, repressed, and all genes, respectively. The trend lines are based on local polynomial regression fitting, by excluding data points below the 2nd and above the 98th percentile of MYC binding variation. For the same set of genes, the Spearman correlation is reported. The vertical dashed lines identify, for each system, the amount of MYC change at which promoters begin to increase their share of MYC binding (see panel *B*). Triangles mark outlier data points (top 0.5% of the data on both the *x*- and *y*-axis) that were forced within the plot range limits; the original values for these data points were used to derive the trend lines and correlation values. (*B*) Schema illustrating the concept of MYC share: despite the overall increase in MYC binding, depicted by the increased peaks area, the proportion of MYC binding out of total MYC-bound (the share, reported as %) could either increase or decrease. (*C*) Receiver operating characteristic (ROC) curves for the ability of discriminating induced and repressed genes at growing thresholds of changes in MYC binding (see Methods for details). (AUC) Area under the curve. For each system, the dot corresponds to the variation of MYC at which promoters begin increasing their share of MYC binding. These identified the best trade-off between sensitivity and specificity, thus maximizing the ability to simultaneously classify induced and repressed genes.

Previous analyses in U2OS^tet-MYC^ cells indicated that the extent of either activation or repression by overexpressed MYC correlated with the increase in MYC occupancy at promoters (Walz et al. 2014). Our re-analysis of the same data confirmed this observation, whether performed as originally described (i.e., by binning activated and repressed mRNAs) (Supplemental Fig. S1A, top) or considering every differentially expressed transcript (Fig. 1A). The four other model systems confirmed the positive correlation between gene activation and binding (Fig. 1A; Supplemental Fig. S1A, red lines) but showed the opposite for repressed genes (blue lines): as a consequence, the whole transcriptome showed a more homogeneous trend, repressed and activated genes showing the lowest and highest gains in MYC binding, respectively (Fig. 1A, orange lines). This relationship was reinforced by calculating the “share” of MYC at each promoter, obtained by normalizing each binding event by the total amount of MYC associated with the genome. The share concept was adopted to focus on the changes in binding that were emerging from the global changes (which were normalized out). This concept allowed us to define two classes of promoters with either increased or decreased shares following MYC activation (Fig. 1B): remarkably, the threshold separating these two classes (Fig. 1A, dashed vertical lines) identified the amount of MYC binding that optimally separated transcriptionally activated from repressed genes in each model system (Fig. 1A,C). This effect was further reinforced when distinguishing primary (MYC-dependent) from secondary (independent) regulatory events in tet-MYC liver tumors (Supplemental Fig. S1B,C; Kress et al. 2016).

The apparently opposite regulatory behavior of repressed genes in U2OS cells is a paradoxical result, which remains to be explained. We deem it unlikely, however, for this to represent a real distinctive feature of these cells. Indeed, we must note that the negative correlation between the changes in MYC share and transcription of repressed genes in the U2OS data set was remarkably low and not statistically significant, whether analyzing genes individually (Spearman correlation −0.03, *P* = 0.12) (Fig. 1A) or binned (−0.05, *P* = 0.81) (Supplemental Fig. S1A). The other data sets, instead, all gave robust positive and statistically significant correlations. The reasons for this discrepancy may reside in a number of technical parameters, the resolution of which is beyond the scope of our work.

In U2OS^tet-MYC^ cells, the transcriptional response to MYC has also been correlated with the MYC/ZBTB17 ratio at promoters (Lorenzin et al. 2016): indeed, we confirmed that the MYC/ZBTB17 ratio and the variation in the MYC share were complementary predictors of gene expression changes (Supplemental Fig. S1E). In 3T9^MYC-ER^ cells and Eµ-*myc* lymphomas, instead, the MYC share had much stronger predictive values, with minor albeit significant contributions of ZBTB17—whether expressed as a MYC/ZBTB17 ratio or as straight ZBTB17 binding (Supplemental Fig. S1E). In particular, the ability of MYC to recruit RNAPII seemed to be fine-tuned by ZBTB17: indeed, in all the systems analyzed, the gain in MYC binding that discriminated between activation and repression slightly increased with the level of ZBTB17 binding (Supplemental Fig. S1F). Overall, the above observations show that the variation in MYC share at promoters was the main feature separating induced from repressed genes across cell types: gains in MYC binding were roughly proportional to gene activation, as previously reported (Walz et al. 2014; Lorenzin et al. 2016), while the effect was reversed for repressed promoters.

Another feature noted in the U2OS^tet-MYC^ model was that the relative affinities of promoters for MYC counter-correlated with the gains in binding and hence with the magnitude of the transcriptional response upon MYC activation (Lorenzin et al. 2016). In particular, high-affinity promoters were prebound the most efficiently by endogenous MYC in control cells and showed minimal gains in binding, while low-affinity promoters showed little prebound MYC and the strongest gains: indeed, our analyses confirmed this observation in the five cell types (Supplemental Fig. S1D). However, very few promoters showed actual saturation by MYC in control cells, including proliferating U2OS^tet-MYC^ cells (Lorenzin et al. 2016), since most promoters—even among those with the highest affinities—showed significant gains with exogenous MYC (Supplemental Fig. S1D). It is noteworthy here that the gain in MYC binding for the high-affinity promoters resulted in the maintenance of their MYC share, consistent with their weak or absent transcriptional response (Fig. 1A).

### Alterations of transcriptional dynamics upon MYC activation

**Figure 2.**
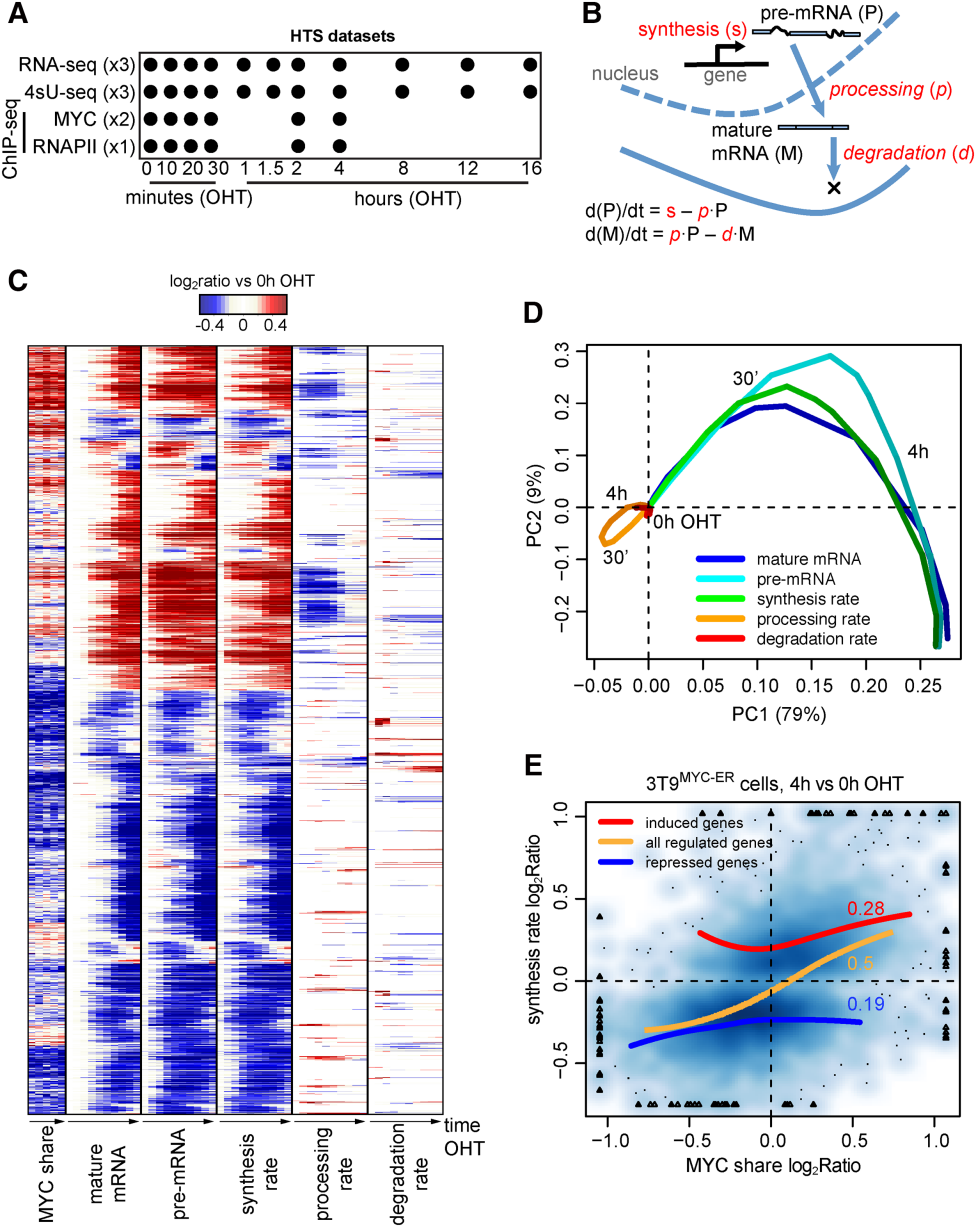
Dynamics of mRNA synthesis, processing, and degradation. (*A*) Study design indicating the collected HTS data, the time points of OHT treatment, and the number of replicates. In 4sU-seq samples, 4sU was added to the culture medium for 10 min prior to collection at every time point, to label and purify newly synthesized RNA. (*B*) Schematic representation of the kinetic rates (in red in the figure) of transcriptional regulation: immature and mature mRNA abundances, as well as synthesis rates, were derived directly from the experimental data, taking advantage of exonic and intronic reads in total and nascent RNA-seq data. Conversely, processing and degradation rates (in italic in the figure) were inferred from the integrated analysis of these data based on mathematical modeling. (*C*) Hierarchical clustering of the transcriptional response and change of MYC share for MYC-bound differentially expressed genes. Genes and time points are depicted in the rows and columns, respectively, and up- (red) or down- (blue) modulation is determined as the log_2_ ratio to the untreated condition. (*D*) Principal component analysis of the transcriptional response depicted in *C*; for each data type, subsequent time points of OHT treatment follow from light to darker shading; PC1 and PC2 are the first and second principal components, accounting for 80% and 9% of the explained variance, respectively. (*E*) Density scatter plot (darker colors for higher density) of the variation of MYC ChIP-seq signal within promoters (*x*-axis) vs. the corresponding change in synthesis rate (*y*-axis). The red, blue, and orange lines capture the trend for induced, repressed, and both (combined) set of genes, respectively. For the same set of genes, the Spearman correlation is reported.

Having determined the relationship between MYC binding and transcriptional responses, we undertook a detailed kinetic analysis of mRNA dynamics in 3T9^MYC-ER^ cells. As a preliminary control, we confirmed the association of the MYC-ER protein with chromatin within 10 min of OHT treatment (Supplemental Fig. S2A), accompanied by an immediate increase in the immature forms of known MYC-induced mRNAs, followed by accumulation of the mature forms at later time points (Supplemental Fig. S2B,C). We thus used RNA-seq to profile both total and newly synthesized RNA (the latter isolated after 10 min of metabolic labeling with 4sU) (Melvin et al. 1978; Rabani et al. 2011) in a detailed time-course following OHT stimulation (Fig. 2A). Integrative analysis of nascent and total RNA-seq time-courses was performed with INSPEcT, a tool that we previously developed (de Pretis et al. 2015) to determine the rates of RNA synthesis (i.e., transcription), processing, and degradation (henceforth, “kinetic rates”) (Fig. 2B). Briefly, mathematical modeling of the intronic and exonic signal from both nascent and total RNA-seq data allowed us to derive the rates of pre-mRNA synthesis and their change along the time-course, followed by the estimation of the rates of pre-mRNA processing and mature mRNA degradation (see Methods for details). In total, 4909 MYC-bound genes showed altered kinetic rates following MYC-ER activation: changes in synthesis were the most prevalent, occurred at a large majority of these loci (4651, or 95%), and accounted for the variations in mature and pre-mRNA levels (Fig. 2C,D). Effects on RNA degradation and processing were less prevalent (976 and 1333 loci, respectively) (Fig. 2C,D). ChIP-seq analysis of MYC binding at early time points (Fig. 2A) revealed that changes in synthesis rate were generally paralleled by changes in the MYC share at promoters (Fig. 2C,E), confirming the correlation between MYC share and gene regulation (Fig. 1).

### MYC regulates transcription primarily through RNAPII loading

**Figure 3.**
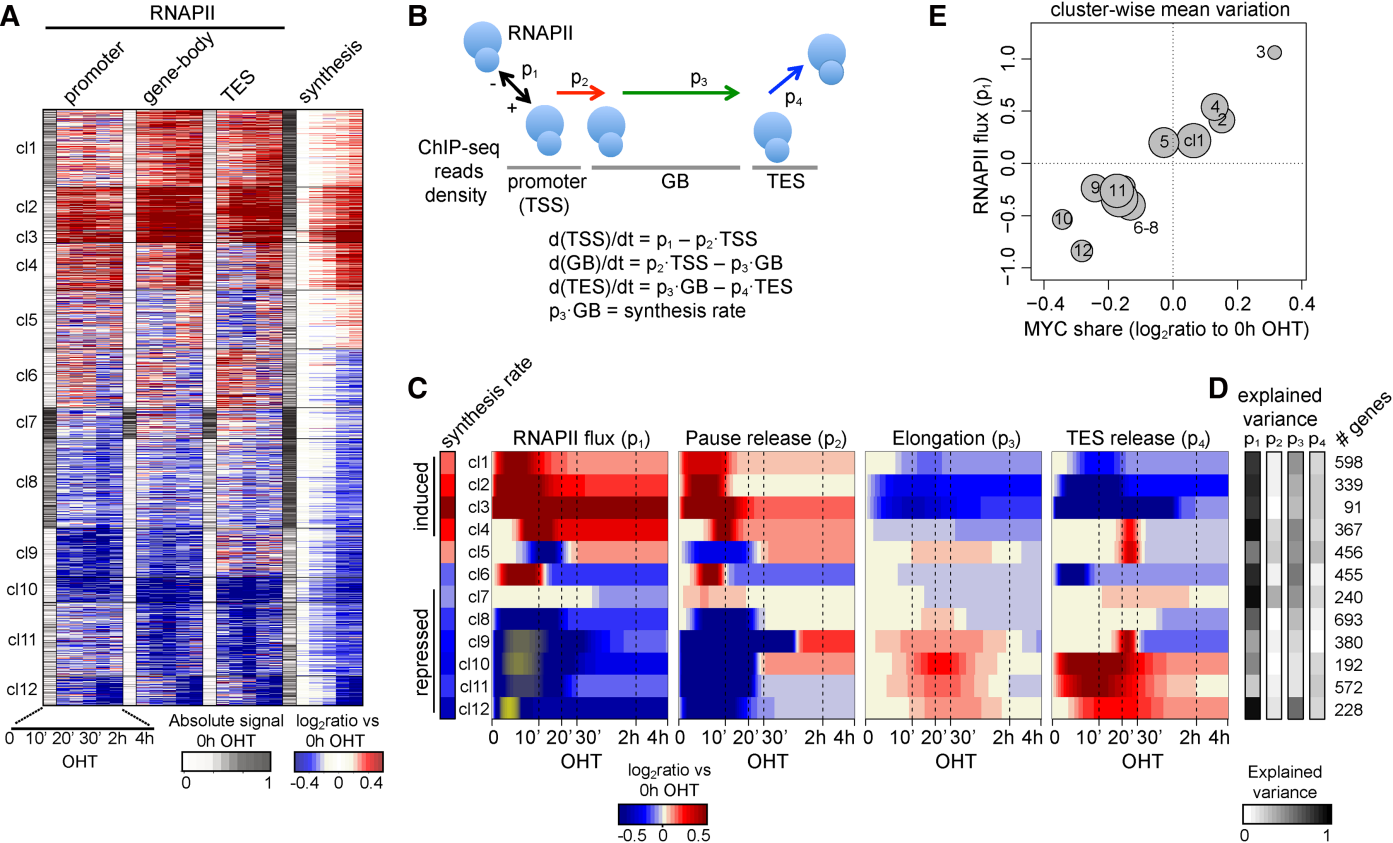
Dynamics of RNAPII loading and progression along transcriptional units. (*A*) Clusters grouping genes with similar modulation of RNAPII density (change in ChIP-seq reads within various portions of the transcriptional units) and synthesis rate; for each data type, the first column (white to gray) indicates the normalized intensity of the feature in the untreated condition, while the other columns indicate the up- (red) or down- (blue) modulation as the log_2_ ratio to the untreated condition. (*B*) The model used to describe progression of RNAPII through the transcriptional units. p_1_ is the flux of polymerase into the promoter region, p_2_ is the rate of release from promoter, p_3_ the elongation rate, and p_4_ the rate of release from the TES. Model parameters were inferred from the variation of RNAPII density over time within the promoter, gene-body, and TES, respectively. The net amount of RNAPII within each region of a gene was assumed to result from its entry from the preceding compartment and its exit to the next one—the compartment before the promoter and after the TES being the nucleoplasm. (*C*) Heat map of the changes in p_1–4_ for each cluster (rows), determined as the log_2_ ratio to the untreated condition (red for increase, and blue for decrease). Yellow for p_1_ marks negative parameter values, i.e., loss of RNAPII from the promoters. (*D*) For each cluster, the explained variance of a model where only that parameter can vary over time is displayed, indicating the ability to recapitulate the changes in RNAPII binding and synthesis rate; the number of genes is indicated on the right. (*E*) Variation of MYC share at promoters (*x*-axis, determined for each cluster as the average change at 10 min–4 h OHT compared to the untreated condition) versus the variation of the RNAPII flux (*y*-axis, at 4 h OHT); the size of the circle is proportional to the number of genes within the cluster.

To further address the mechanisms underlying MYC-regulated transcription, we also mapped RNAPII by ChIP-seq (Fig. 2A). We then computed the variations in RNAPII levels in promoters, gene bodies, and transcription end sites and used these values—alongside the variations in RNA synthesis—to cluster the 4651 differentially transcribed genes: this partitioned our data set into 14 clusters (two small clusters comprising only 13 and 27 genes were removed from subsequent analyses) (Fig. 3A; Supplemental Fig. S3A). For each cluster, we modeled the different steps involved in RNAPII-mediated transcription, based on four parameters (Fig. 3B; Jonkers and Lis 2015): the flux of RNAPII at the promoter (p_1_, net amount of recruited and lost polymerase per hour), the rate of RNAPII pause-release from the promoter (p_2_), the elongation rate (p_3_, modeled as the ratio between the measured synthesis rate and RNAPII density over the gene-body) (Danko et al. 2013), and the rate of release from the transcription end site (TES; p_4_). Briefly, the changes in density of RNAPII in each region of the transcriptional unit are considered as the net result of (1) the flux of incoming RNAPII from the upstream region, and (2) the flux of RNAPII exiting to the downstream region, both governed by the corresponding kinetic rate (see Methods for details). Importantly, the four parameters (p_1_–p_4_), inferred along the entire time-course for each cluster (Fig. 3C; Supplemental Fig. S3B), accurately recapitulated the experimental data (Supplemental Fig. S3C,D).

Previous studies suggested that MYC activates transcription mainly—if not solely—at the level of RNAPII pause-release (p_2_), rather than loading (p_1_) (Rahl et al. 2010). In apparent agreement with this concept, MYC activation resulted in increased pause-release at activated promoters (clusters cl1–4, with the exception of cl5) (Fig. 3C; Supplemental Fig. S3B). However, these changes were associated with sudden and more prominent changes in RNAPII loading (Fig. 3A,C). Following this initial burst, recruitment rates were stabilized at higher levels compared to the untreated condition (Supplemental Fig. S3B). We conclude that MYC promotes both RNAPII loading and pause-release, thus regulating two key steps in transcription initiation (Jonkers and Lis 2015).

In parallel with the above, down-regulated clusters (cl7–12, with the exception of cl6) showed marked decreases in the RNAPII signal at promoters (Fig. 3A), owing primarily to decreased loading (p_1_) (Fig. 3C; Supplemental Fig. S3B). In four clusters (cl9–12), p_1_ values transiently became negative, indicating nonproductive detachment of RNAPII from promoters. Importantly, the reduced levels of RNAPII were not due to increased pause release, as p_2_ values also rapidly went down in response to MYC activation (with the exception of cl7). Thus, MYC-repressed genes underwent a general reduction in RNAPII loading.

We showed above that changes in MYC share at promoters correlated with changes in gene expression (Fig. 1) and synthesis rates (Fig. 2C,E). Logically, RNAPII flux at promoters also followed the MYC share (Fig. 3E) and was sufficient to explain most of the variation in polymerase occupancy and synthesis rate (p_1_, on average 69% of explained variance) (Fig. 3D), while p_2_, p_3_, and p_4_ showed lesser relevance (10%, 34%, and 17%, respectively). Hence, the first effect of MYC-ER was a rapid modulation of RNAPII flux at promoters, with increases in recruitment at activated genes and, reciprocally, decreases—or even inversion—at repressed loci, which also showed the lowest gains in MYC binding. Altogether, the above observations show that acute activation of MYC causes direct recruitment of RNAPII to activated promoters, accounting for most of the observed changes in RNA synthesis, at either up- or down-regulated loci.

### Dynamics of RNAPII elongation and pre-mRNA processing

The above observations revealed a paradox, in that changes in synthesis rate were delayed relative to the variations in RNAPII density at gene-bodies. In particular, RNAPII accumulated within induced genes and, reciprocally, was already lost from repressed genes 10 min after MYC-ER activation (Fig. 3A), originating at the 5′ end of transcriptional units (Supplemental Fig. S4A). Notably, at induced loci, the increases in RNAPII density exceeded those in RNA synthesis (Supplemental Fig. S3C), suggesting that decreasing rates of polymerase elongation contributed to RNAPII accumulation in induced genes (Ehrensberger et al. 2013), as confirmed by our modeling (Fig. 3C). Indeed, fixing elongation rates in silico at the levels of the untreated condition was detrimental for the ability of the model to predict either RNAPII dynamics at gene-bodies or synthesis rates (Supplemental Fig. S4B). Moreover, while MYC-induced increases in pause-release also contributed to RNAPII accumulation in induced genes, the changes in elongation were more sustained (Fig. 3C) and contributed a larger part of the variance (p_2_ vs. p_3_) (Fig. 3D). This situation was mirrored at repressed loci, at which changes in p_2_ and p_3_ determined a reduced density of RNAPII at the gene-body, especially for the strongest responders (cl9–12) (Fig. 3A,C).

**Figure 4.**
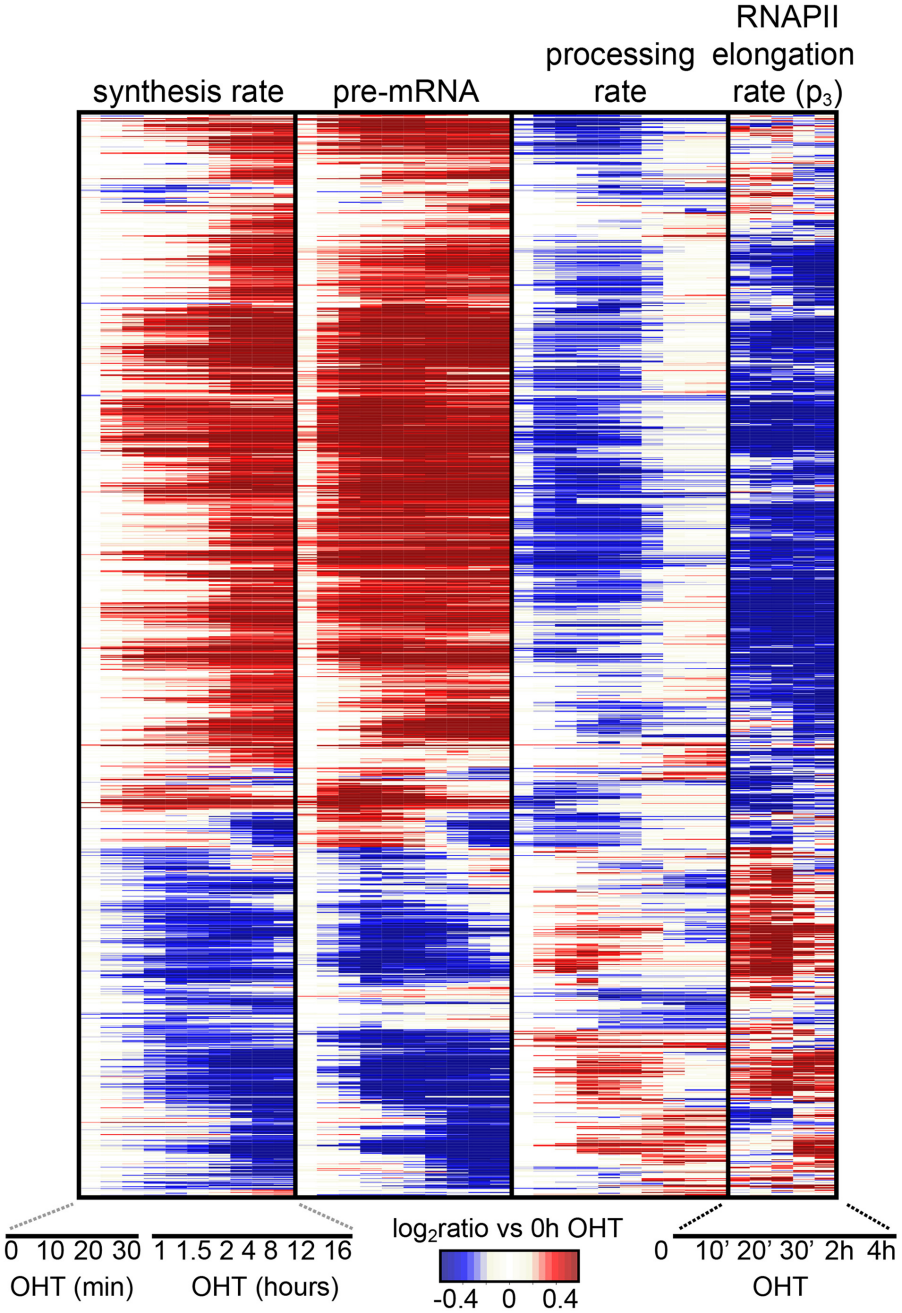
Dynamics of RNAPII elongation and pre-mRNA processing. Hierarchical clustering of synthesis rates, pre-mRNA concentrations, processing rates, and RNAPII elongation rates for the subset of MYC-bound differentially expressed genes having variable processing rate (1333 genes). Genes and time points are depicted in the rows and columns, respectively, and up- (red) or down- (blue) modulation is determined as the log_2_ ratio to the untreated condition.

Shortly after the decrease in RNAPII elongation, almost half of the induced genes showed decreasing rates of pre-mRNA processing with concomitant accumulation of pre-mRNA, anticipating the transcriptional response (Fig. 4; Supplemental Fig. S4C). Opposite effects occurred at a subset of repressed genes. In silico modeling confirmed that fixing the processing rates of the induced genes at the levels of the untreated condition prevented the observed accumulation of pre-mRNA (Supplemental Fig. S4D,E). Consistent with previous observations (Dujardin et al. 2014), the observed changes in RNAPII elongation were associated also with alterations in splicing affecting exon incorporation or intron retention at several hundred loci (Supplemental Fig. S4F–I). These alterations peaked at the time of maximum changes in the rates of elongation and processing (30 min and 1 h for induced and repressed, respectively) and were enriched at the 3′ end of genes (Supplemental Fig. S4G,I). Additional alterations occurred late, possibly due to the downstream action of splicing factors, which were enriched among MYC-regulated gene products.

## Discussion

Altogether, our analysis reveals several key features of the transcriptional alterations brought about by MYC overexpression in various systems. First, we quantified the ability of MYC binding to predict the transcriptional response of its target genes, revealing that the variation in the proportion of MYC bound at promoters (MYC share) could effectively discriminate induced from repressed genes. The role of the MYC share was examined in various systems, including models with global increases in transcriptional activity (or transcriptional amplification; P493-6 and Eµ-*myc*) (Lin et al. 2012; Nie et al. 2012), or without (U2OS^tet-MYC^ and 3T9^MYC-ER^). The role of the MYC share in directing the induction or repression of MYC target genes emerged as a common denominator among these systems, strengthening the view that transcriptional amplification, when observed, does not occur as a direct effect of MYC but rather as a secondary consequence of cellular activation (Kress et al. 2016).

Second, we dissected the effects of MYC on the RNAPII life cycle along transcriptional units, identifying loading as the key regulated step. Previous reports indicated that MYC activates transcription through RNAPII pause-release (Eberhardy and Farnham 2002; Bouchard et al. 2004; Rahl et al. 2010): while we confirmed this effect at MYC-activated genes, pause-release was secondary to RNAPII loading. It is noteworthy here that we are not the first to report that MYC binding leads to increased RNAPII at promoters (Walz et al. 2014): our modeling, however, allowed us to directly quantify this step, overcoming the limitations of using solely ChIP-seq density data (Ehrensberger et al. 2013).

Third, transcriptional repression was associated both with the lowest gains in MYC binding and with decreased RNAPII recruitment. As the amount of chromatin-bound RNAPII remained relatively stable following MYC activation (Supplemental Fig. S3E), loss of RNAPII from repressed promoters may have largely resulted from competition for limiting amounts of polymerase. Thus, besides interfering with the activating function of ZBTB17 or other transcription factors, MYC may repress transcription indirectly, owing to passive loss of RNAPII. MYC may also elicit repression through other indirect mechanisms, such as the induction of PTEN, resulting in augmented activity of the Polycomb Repressive Complex PRC2 (Kaur and Cole 2013; Cole 2014): this mechanism might not be relevant in our experiments, however, as PTEN was induced in none of the systems analyzed here.

Fourth, and consistent with the above, transcriptional repression by MYC is partly independent from its interaction with ZBTB17. Indeed, the strongest predictor of transcriptional outcome was the MYC share at promoters, more significant than either ZBTB17 binding or the MYC/ZBTB17 ratio (Walz et al. 2014). Moreover, a MYC mutant defective in ZBTB17 binding (MYC V394D, or VD) retained both activating and repressing activities in the tet-MYC liver model (Kress et al. 2016), albeit slightly less effective than wild-type MYC (Supplemental Fig. S1G). Our results also support the concept that ZBTB17, rather than forming repressive complexes with MYC at repressed promoters, may affect its ability to recruit RNAPII at those loci.

Fifth, the rapid effect of MYC-ER on RNAPII recruitment probably caused an overload of the transcriptional machinery at induced loci, as revealed by reduced rates of RNAPII elongation and accumulation of RNAPII at gene-bodies. This could be due to the combination of several factors, including RNAPII crowding, shortage of nucleotides, or the interplay between RNAPII and the spliceosome. In particular, a role for the latter is supported by our data, since pre-mRNA processing rates decreased in parallel to the increase of RNAPII at gene-bodies, leading to the accumulation of unprocessed mRNAs. This points to a limiting activity of the spliceosomal machinery at MYC-activated loci, consistent with its critical role for cell survival in MYC-driven tumors (Hsu et al. 2015; Koh et al. 2015).

## Methods

### Cell culture

3T9^MYC-ER^ fibroblasts were obtained by infecting 3T9 *c-Myc* flox/flox immortalized fibroblasts (Trumpp et al. 2001) with a pBabe-Bleo retrovirus encoding the MYC-ER chimera (Sabò et al. 2014) and were cultured in DMEM medium, supplemented with 10% fetal bovine serum, 2 mM L-glutamine, and penicillin/streptomycin. These cells are not listed in any database of commonly misidentified cells and are negative for mycoplasma. Cells were used at subconfluent cell densities for all experiments. Due to infection with the pBabe bleomycin *MYC-ER* construct, cells are resistant for zeocin. Upon thawing, cells were maintained for 7–10 d in zeocin-containing medium (100 µg/mL) but grown without zeocin for subsequent experiments. MYC-ER activation was achieved by addition of 400 nM of the synthetic 4-hydroxytamoxifen (OHT; Sigma-Aldrich). For RNA degradation validation, cells were treated with 1 µM flavopiridol hydrochloride hydrate (Sigma-Aldrich) for the indicated time points.

### 4sU RNA-seq

Detection of nascent RNA by metabolic labeling using 4sU (4-methio-uridine) has been performed as described before (Sabò et al. 2014) by labeling the cells with 300 mM 4sU for 10 min. The 4sU-sequencing libraries were prepared with the TruSeq RNA Sample Prep kit v2 (Illumina) following the manufacturer's instructions starting from the RNA fragmentation step.

## Data access

The high-throughput sequencing data generated in this study have been submitted to the NCBI Gene Expression Omnibus (GEO; http://www.ncbi.nlm.nih.gov/geo/) under accession number GSE98420. The complete source code for all analyses, including intermediary results and the raw images, is available in the Supplemental Material.

## Supplementary Material

Supplemental Material

## References

[DEPRETISGR226035C1] Bouchard C, Marquardt J, Brás A, Medema RH, Eilers M. 2004 Myc-induced proliferation and transformation require Akt-mediated phosphorylation of FoxO proteins. EMBO J 23: 2830–2840. 15241468 10.1038/sj.emboj.7600279 PMC514943

[DEPRETISGR226035C2] Braun KA, Young ET. 2014 Coupling mRNA synthesis and decay. Mol Cell Biol 34: 4078–4087. 25154419 10.1128/MCB.00535-14 PMC4248707

[DEPRETISGR226035C3] Cole MD. 2014 MYC association with cancer risk and a new model of MYC-mediated repression. Cold Spring Harb Perspect Med 4: a014316. 24985129 10.1101/cshperspect.a014316 PMC4066640

[DEPRETISGR226035C4] Danko CG, Hah N, Luo X, Martins AL, Core L, Lis JT, Siepel A, Kraus WL. 2013 Signaling pathways differentially affect RNA polymerase II initiation, pausing, and elongation rate in cells. Mol Cell 50: 212–222. 23523369 10.1016/j.molcel.2013.02.015 PMC3640649

[DEPRETISGR226035C5] de Pretis S, Kress T, Morelli MJ, Melloni GEM, Riva L, Amati B, Pelizzola M. 2015 INSPEcT: a computational tool to infer mRNA synthesis, processing and degradation dynamics from RNA- and 4sU-seq time course experiments. Bioinformatics 31: 2829–2835. 25957348 10.1093/bioinformatics/btv288

[DEPRETISGR226035C6] Dujardin G, Lafaille C, la Mata de M, Marasco LE, Muñoz MJ, Le Jossic-Corcos C, Corcos L, Kornblihtt AR. 2014 How slow RNA polymerase II elongation favors alternative exon skipping. Mol Cell 54: 683–690. 24793692 10.1016/j.molcel.2014.03.044

[DEPRETISGR226035C7] Eberhardy SR, Farnham PJ. 2002 Myc recruits P-TEFb to mediate the final step in the transcriptional activation of the cad promoter. J Biol Chem 277: 40156–40162. 12177005 10.1074/jbc.M207441200

[DEPRETISGR226035C8] Ehrensberger AH, Kelly GP, Svejstrup JQ. 2013 Mechanistic interpretation of promoter-proximal peaks and RNAPII density maps. Cell 154: 713–715. 23953103 10.1016/j.cell.2013.07.032

[DEPRETISGR226035C9] Hsu TYT, Simon LM, Neill NJ, Marcotte R, Sayad A, Bland CS, Echeverria GV, Sun T, Kurley SJ, Tyagi S, 2015 The spliceosome is a therapeutic vulnerability in MYC-driven cancer. Nature 525: 384–388. 26331541 10.1038/nature14985 PMC4831063

[DEPRETISGR226035C10] Jonkers I, Lis JT. 2015 Getting up to speed with transcription elongation by RNA polymerase II. Nat Rev Mol Cell Biol 16: 167–177. 25693130 10.1038/nrm3953 PMC4782187

[DEPRETISGR226035C11] Kaur M, Cole MD. 2013 MYC acts via the PTEN tumor suppressor to elicit autoregulation and genome-wide gene repression by activation of the Ezh2 methyltransferase. Cancer Res 73: 695–705. 23135913 10.1158/0008-5472.CAN-12-2522 PMC3549058

[DEPRETISGR226035C12] Koh CM, Bezzi M, Low DHP, Ang WX, Teo SX, Gay FPH, Al-Haddawi M, Tan SY, Osato M, Sabò A, 2015 MYC regulates the core pre-mRNA splicing machinery as an essential step in lymphomagenesis. Nature 523: 96–100. 25970242 10.1038/nature14351

[DEPRETISGR226035C13] Kress TR, Sabò A, Amati B. 2015 MYC: connecting selective transcriptional control to global RNA production. Nat Rev Cancer 15: 593–607. 26383138 10.1038/nrc3984

[DEPRETISGR226035C14] Kress TR, Pellanda P, Pellegrinet L, Bianchi V, Nicoli P, Doni M, Recordati C, Bianchi S, Rotta L, Capra T, 2016 Identification of MYC-dependent transcriptional programs in oncogene-addicted liver tumors. Cancer Res 76: 3463–3472. 27197165 10.1158/0008-5472.CAN-16-0316

[DEPRETISGR226035C15] Lin CY, Lovén J, Rahl PB, Paranal RM, Burge CB, Bradner JE, Lee TI, Young RA. 2012 Transcriptional amplification in tumor cells with elevated c-Myc. Cell 151: 56–67. 23021215 10.1016/j.cell.2012.08.026 PMC3462372

[DEPRETISGR226035C16] Lorenzin F, Benary U, Baluapuri A, Walz S, Jung LA, Eyss von B, Kisker C, Wolf J, Eilers M, Wolf E. 2016 Different promoter affinities account for specificity in MYC-dependent gene regulation. eLife 5: 1009. 10.7554/eLife.15161 PMC4963202 27460974

[DEPRETISGR226035C17] Melvin WT, Milne HB, Slater AA, Allen HJ, Keir HM. 1978 Incorporation of 6-thioguanosine and 4-thiouridine into RNA. Application to isolation of newly synthesised RNA by affinity chromatography. Eur J Biochem 92: 373–379. 570106 10.1111/j.1432-1033.1978.tb12756.x

[DEPRETISGR226035C18] Nie Z, Hu G, Wei G, Cui K, Yamane A, Resch W, Wang R, Green DR, Tessarollo L, Casellas R, 2012 c-Myc is a universal amplifier of expressed genes in lymphocytes and embryonic stem cells. Cell 151: 68–79. 23021216 10.1016/j.cell.2012.08.033 PMC3471363

[DEPRETISGR226035C19] Rabani M, Levin JZ, Fan L, Adiconis X, Raychowdhury R, Garber M, Gnirke A, Nusbaum C, Hacohen N, Friedman N, 2011 Metabolic labeling of RNA uncovers principles of RNA production and degradation dynamics in mammalian cells. Nat Biotechnol 29: 436–442. 21516085 10.1038/nbt.1861 PMC3114636

[DEPRETISGR226035C20] Rahl PB, Lin CY, Seila AC, Flynn RA, McCuine S, Burge CB, Sharp PA, Young RA. 2010 c-Myc regulates transcriptional pause release. Cell 141: 432–445. 20434984 10.1016/j.cell.2010.03.030 PMC2864022

[DEPRETISGR226035C21] Sabò A, Kress TR, Pelizzola M, de Pretis S, Gorski MM, Tesi A, Morelli MJ, Bora P, Doni M, Verrecchia A, 2014 Selective transcriptional regulation by Myc in cellular growth control and lymphomagenesis. Nature 511: 488–492. 25043028 10.1038/nature13537 PMC4110711

[DEPRETISGR226035C22] Trumpp A, Refaeli Y, Oskarsson T, Gasser S, Murphy M, Martin GR, Bishop JM. 2001 c-Myc regulates mammalian body size by controlling cell number but not cell size. Nature 414: 768–773. 11742404 10.1038/414768a

[DEPRETISGR226035C23] Walz S, Lorenzin F, Morton J, Wiese KE, Eyss von B, Herold S, Rycak L, Dumay-Odelot H, Karim S, Bartkuhn M, 2014 Activation and repression by oncogenic MYC shape tumour-specific gene expression profiles. Nature 511: 483–487. 25043018 10.1038/nature13473 PMC6879323

