# Supplementary material for: Integrative analysis of RNA polymerase II and transcriptional dynamics upon MYC activation
Source: Genome Res. 2017 Oct;27(10):1658–64. doi: 10.1101/gr.226035.117 (PMC5630029; doi:10.1101/gr.226035.117)
Supplement: Supplemental Material [file supp_27_10_1658__index.html]

Integrative analysis of RNA polymerase II and transcriptional dynamics upon MYC activation — Supplemental Material 

# Integrative analysis of RNA polymerase II and transcriptional dynamics upon MYC activation

## Supplemental Material

- Supplemental\_Methods.docx
- Supplemental\_Table\_S1.xlsx
- Supplemental\_Code.zip
- Supplemental\_Figures.docx
