# Supplementary material for: Integrative analysis of RNA polymerase II and transcriptional dynamics upon MYC activation
Source: Genome Res. 2017 Oct;27(10):1658–64. doi: 10.1101/gr.226035.117 (PMC5630029; doi:10.1101/gr.226035.117)
Supplement: Supplemental Material [file supp_gr.226035.117_Supplemental_Methods.docx]

**Supplemental Methods**

**Integrative analysis of RNA Polymerase II and transcriptional dynamics upon MYC activation**

Stefano de Pretis, Theresia R. Kress, Marco J. Morelli, Arianna Sabò, Chiara Locarno, Alessandro Verrecchia, Mirko Doni, Stefano Campaner, Bruno Amati, Mattia Pelizzola

**Index of Supplemental Methods**

1. Experimental methods
   1. Subcellular fractionation
   2. RNA extraction, qRT-PCR and RNA-seq
   3. Chromatin immunoprecipitation
   4. Primer design and list of primers
2. ChIP-seq data analysis
3. Predictive power of ChIP-seq features over RNA expression (ROC plots)
4. Dynamics of transcription following MYC-ER activation (Figures 1 and 2)
   1. Number of samples
   2. Analysis of total and nascent RNA-seq data, and determination of the kinetic rates
   3. Analysis of the transcriptional response to MYC-ER activation
5. Dynamics of RNAPII loading and progression through transcriptional units (Figure 3)
6. Dynamics of RNAPII elongation and pre-mRNA processing (Figure 4)
7. References
8. **Experimental Methods**

1. Subcellular fractionation

Cells were biochemically fractionated according to a previously published protocol (Smale et al. 2012), with all steps performed on ice using ice-cold buffers. Briefly, 3T9^MYC-ER^ fibroblasts were trypsinized and pelleted (1,500 rpm, 5 min, 4°C). Cells were resuspended in 300µl of lysis buffer 1(10 mM Tris-HCl pH 7.5, 150 mM NaCl, 0.4% (v/v) NP-40), incubated for 5 mins and then put on top of a 750µl sucrose cushion (24% w/v in cytoplasmic lysis buffer) and centrifuged (13,000 rpm, 10 min, 4°C). The supernatant was kept as soluble fraction. The remaining pellet was resuspended in 300µl glycerol buffer (20mM Tris-HCl pH8.0, 75mM NaCl, 0.5 mM EDTA, 50% (v/v) glycerol, 0.85 mM DTT) and 300µl of lysis buffer 2 (20 mM HEPES pH 7.6, 7.5 mM MgCl_2_, 0.2 mM EDTA, 300 mM NaCl, 1M Urea, 1% (v/v) NP-40, 0.1 mM DTT) was added. After gentle vortexing, and incubation for 5 mins sample were pelleted again (13,000 rpm, 2 min, 4°C). The supernatant was retained as Wash (mainly nuclear soluble components). The pellet was washed in glycerol/nuclei lysis buffer, pelleted and resuspended in PBS to obtain the chromatin-associated fraction. Protein fractions were subjected to SDS-Page, transferred onto PVDF membranes (Millipore) and detected using the following antibodies: c-Myc Y69 (ab32072, Abcam), Vinculin (V9264, Sigma), histone H3 (ab1791, Abcam), RNAP2 (sc-899, Santa Cruz), HRP-conjugated goat-anti-rabbit IgG (170-6515, Biorad) and goat-anti-mouse IgG (170-6516, Biorad). Chemiluminescence was detected using X-ray films (Figure S2a) or a CCD camera, ChemiDoc System, Bio-Rad (Figure S3e).

1. RNA extraction, qRT-PCR and RNA-seq

Total RNA from 3T9^MYC-ER^ fibroblasts was purified using miRNeasy Mini columns (Qiagen) according to the manufacturer's protocol, including the on-column DNase I digestion step. cDNA of 1µg was transcribed using the reverse transcriptase ImPromII (Promega) and oligodT and random primers. In experiments assessing pre-mRNA levels, only random primers were used. 10ng of cDNA per reaction were used for quantitative reverse transcriptase PCR (qRT-PCR) analyses, amplified with the FAST SYBR Green Master Mix and quantified using the 7500 Fast Real-Time PCR machine (both Applied Biosystems).

For RNA-sequencing, 5µg total RNA (RNA integrity number ≥ 7.5) were treated with Ribozero rRNA removal kit (Epicentre) and purified on RNeasy MinElute columns (Qiagen). RNA integrity (see above) and successful removal of ribosomal RNA were assessed with the Agilent 2100 Bioanalyser (Agilent Technologies). RNAseq libraries were prepared with the TruSeq RNA Sample Prep Kit v2 (Illumina) following the manufacturer’s protocol starting from the RNA fragmentation step.

1. Chromatin immunoprecipitation

Was performed as described in (Sabò et al. 2014). In particular, the RNA Pol II holoenzyme complex was detected with the RNAPII N20 (Santa Cruz, sc-899) antibody directed against the Polr2a subunit.

1. Primer design and list of primers

The following primers for pre-mRNA and mature mRNA expression (Figures S2b and S2c) were designed with help of computer-assisted primer design software (Universal ProbeLibrary, Roche; Primer3):

- TGGAGGTTGTTTTGTTGCTG (forward primer in the intron 1 for the immature form of *Slc16a1*) and ATCGCAGGTGGCATTTTAAG (reverse primer in the exon 2 for the immature form of *Slc16a1*).
- GGATATCATCTATAATGTTGGCTGTC and GCTGCCGTATTTATTCACCAA (for the mature form of *Slc16a1*).
- TGTTTGGGTTTCTTCTCTCACA (forward primer in the intron 1 for the immature form of *2610024G14Rik*) and GTCGGGTTGCAAATAGGAAA (reverse primer in the exon 2 for the immature form of *2610024G14Rik*).
- AGACGCCCACTGGTTTTATG and TGGCTTGAGGACAAAGGTTC (for the mature form of *2610024G14Rik*).

1. **ChIP-seq data analysis**

*Alignment of HTS ChIP-seq data*

Reads were filtering with fastx toolkit version 0.0.13.2, using “fastq_quality_trimmer -Q33 -t 20 -l 10 -v -i” and “fastq_masker -Q33 -q 20 -r -N -v -i”. Reads were then aligned to the reference mouse genome (NCBI37, mm9) using the bwa aligner (version 0.6.2-r126) with default settings.

*Peaks calling*

ChIP-seq narrow peaks were called against the input using MACS2 software (Feng et al. 2012; Zhang et al. 2008) (version 2.0.9, p-value threshold = 1e-5, --mfold=7,30). For RNAPII samples, MACS2 was also used to call broad peaks. For the peak calling of ZBTB17 based on the ChIP-seq in 3T9^MYC-ER^ samples we relaxed the p-value threshold to 1e-2, due to the weak signal returned by the 10E2 antibody. We validated the peak calling with this relaxed threshold by comparing the results obtained by us in the 3T9^MYC-ER^ with those published for the MEF cells (Walz et al. 2014) (cells from which the 3T9 derive): 77% of the promoters bound by MYC in the 3T9^MYC-ER^ cells are also bound in the MEF cells, and the reads density in the shared peaks is highly correlated between the two cell types (Spearman correlation 0.69).

*Quantification of the ChIP-seq intensity and determination of the MYC share*

We estimated the scaling factor between ChIP-seq samples and the ChIP-seq input by a linear model built on regions poor of signal and gene annotations, as in those regions the amount of signal recovered is expected to be due to background and to be identical in both the ChIP and the input samples. We quantified the signal in one thousand regions of 10 Kbp without peaks or annotations and choose the normalization factor (*input_scale*) that minimized the difference between the input and each ChIP sample.

We then estimated the library size (*library_scale*) of each sample by counting reads aligned only within regions rich of signal subtracted of the normalized signal from the input. For MYC ChIP-seq samples, regions rich of signal were identified by the identified peaks. For RNAPII ChIP-seq samples, regions rich of signal were identified as the union of narrow-peaks, broad-peaks, and annotated transcriptional units.

*ChIP-seq signal = (Sample_reads_number - input_reads_number * input_scale) / library_scale*

This definition of library size assumes that the overall amount of protein bound to the genome does not change in different experimental conditions, as confirmed for RNAPII in terms of both the expression of the main subunits and the protein amount (as shown Figure S3e). Rather, MYC binding to the chromatin increases after OHT activation, and the normalization by library_scale returns the proportion of protein bound out of the total on the chromatin, which we called “MYC share”.

For each gene, we quantified the MYC ChIP-signal at promoters regions (defined from 2Kbp upstream to 2Kbp downstream the transcription start site), and we quantified RNAPII in three different regions: the promoter region (defined from 50bp upstream to 700bp downstream the transcription start site), the termination end site region (TES, from 1Kbp upstream to 4Kbp downstream the termination end site) and the gene body (GB, defined as the region between the promoter and TES regions). We used different promoter regions for the quantification of MYC and RNAPII signal because of the different binding patterns of the two proteins in the proximity of the transcription start site. However, the results were not impacted by this choice.

While we used the input samples for the identification of the MYC peaks for all systems, we noticed a remarkably high correlation between input and ChIP-seq data for some of the systems in Figure 1 (U2OS and P493-6 cells, Spearman correlation 0.46 - 0.63) that did not occur in the other systems (0 - 0.29). For this reason, the intensity of MYC binding in Figure 1 is not corrected by the input signal.

1. **Predictive power of ChIP-seq features over RNA expression (ROC plots)**

We used a receiver operating characteristic curve (ROC curve) to measure the predictive power of the changes in MYC or ZBTB17 binding at promoters (*feature*) to the transcriptional response. The ROC curve displays the trade-off between sensitivity and specificity in the context of a binary classification that predict as up- or down-regulated all the genes with values of the *feature* greater or lower than a certain threshold, respectively. We measured the predictive power by measuring the area under the curve (AUC): classifiers with AUC greater than 0.5 perform better than a random choice.

In order to measure the joint contribution of a pair of features (F_1_, F_2_), we built similar classificators to the ones used for single features but using as predictor the linear combination a *F_1_ + b F_2_ + c*. The coefficients were assessed through linear modeling of the log2ratio of the expression (L) given the features under analysis ( *L ~ a F_1_ + b F_2_ + c* ). In the dot-plots of Figures S1f and S1g, the threshold separating induced from repressed genes according to this model is illustrated by a line with equation

*F_2_ = -a/b F_1_ - c/b*

while the value reported on this line indicates the correlation between L and the change in the synthesis rate (the predicted and the observed transcriptional responses, respectively).

1. **Dynamics of transcription following MYC-ER activation (Figures 1 and 2)**
2. Number of samples

The optimal number of time points (10 + the control) and biological replicates (3) necessary for a robust inference of the kinetic rates was determined based on the analyses reported in (de Pretis et al. 2015).

1. Analysis of total and nascent RNA-seq data, and determination of the kinetic rates

*Alignment of HTS RNA-seq data*

Reads were filtered with fastx toolkit version 0.0.13.2, using “fastq_quality_trimmer -Q33 -t 20 -l 10 -v -i” and “fastq_masker -Q33 -q 20 -r -N -v -i”. FASTQ files from both 4sU-seq (nascent mRNAs) and total RNA-seq experiments were first aligned on mouse rRNA sequences obtained from GenBank using the TopHat aligner

(Roberts et al. 2012) with default parameters. Only reads unmapped to ribosomal genes were then aligned on the mouse reference genome (NCBI37, mm9), using the TopHat aligner with default parameters.

*Quantification of raw read counts*

We quantified reads falling on exons and introns for each gene by using the function “makeRPKMs” of the INSPEcT Bioconductor package (de Pretis et al. 2015). Briefly, based on the exon and intron definitions available in the TxDb.Mmusculus.UCSC.mm9.knownGene package this routine builds gene models by aggregating exons from all isoforms of the same gene and considering introns as the gaps between the exons. Reads are mapped to exon features first and then to intron features (solving ambiguities in favor of exons in case of reads mapping to both exon and intron features). Eventually, for each gene, time point of OHT treatment and data type (nascent and total mRNA), the counts of exonic and intronic reads are determined.

*Genes filtering, read counts normalization, and quantification of absolute expression*

We selected 11’513 genes (out of the 18'824 genes with at least one intron) based on their expression (at least 5 read counts in ⅓ of the RNA-seq and 4sU-seq samples). 4sU-seq and RNA-seq samples were normalized using the RUVSeq Bioconductor package (Risso et al. 2014) using a Generalized linear model, thus assuming that the sum of reads mapping to gene features (both exonic and intronic) does not change over time and between replicates - as determined in Figure 3 of (Sabò et al. 2014).

Absolute gene expression was quantified for each gene based on exonic read counts in both 4sU-seq and RNA-seq datasets. For each gene, RUVSeq-normalized read counts were transformed into RPKMs by dividing them by the total length of exons. Considering that the library size effect was already normalized out at the level of read counts (see above), we divided the length normalized counts by the median library size of our dataset. The same procedure was applied to determine the intronic RPKMs.

*Estimation of kinetic rates*

The INSPEcT Bioconductor package (de Pretis et al. 2015) was used to estimate the kinetic rates by mathematical modeling of 4sU-seq and RNA-seq RPKMs, determined for both exons and introns. Briefly, while intronic RPKMs are indicative of pre-mRNA (unspliced) abundance, the exonic RPKMs derive from the sum of mature mRNA (spliced) and pre-mRNA (unspliced) abundances. By integrating the levels of immature and mature mRNAs in both the total and the nascent mRNAs fraction, INSPEcT returns the rates of the three main steps governing the mRNA life-cycle: the rate of synthesis of pre-mRNA molecules (synthesis rate, RPKM/hour), the rate of conversion of pre-mRNA molecules into their mature form (processing rate, 1/hour) and the rate of degradation of the mature RNA molecule (degradation rate, 1/hour). Rates were inferred by considering a 4sU incorporation time of 5 minutes, instead of the experimental labeling time of 10 minutes, following the observation that 4sU incorporation into transcripts starts after 5 minutes of 4sU exposure. Kinetic rates were inferred using the “newINSPEcT” function of the package INSPEcT by setting “totalMedianNorm” to FALSE - considering that RPKMs were previously normalized with RUVSeq - while leaving the other arguments to their default settings.

We used the “modelRates” INSPEcT function to model the kinetic rates over time as impulse models. Following this modeling step, the tool can statistically assess whether the modeling is satisfactory and indicate whether synthesis, processing or degradation rates should be assumed to be constant over time or not. We set the chi-squared threshold to 0.01 for the goodness-of-fit test, and Brown’s method threshold to 0.05 to assess whether kinetic rates are changing over time.

1. Analysis of the transcriptional response to MYC-ER activation

We identified 4’909 genes altered in 3T9^MYC-ER^ cells following MYC-ER activation. This set of genes is the result of the intersection between:

- 11’141 genes with a MYC peak overlapping the promoter region in at least one time point
- 9’439 genes with RPKM of at least 1 for total RNA-seq exons in at least one time point
- 6’415 genes with at least one rate identified as changing by INSPEcT (with either the total or the synthesis rates having an absolute log2 fold change of at least 1.2 compared to the untreated condition).

We used the R implementation (prcomp) of the principal component analysis (PCA) to visualize the responses of mRNA, pre-mRNA and kinetic rates after MYC induction. For each feature, the coordinates of the two principal components were plotted and contiguous time-points were connected by a line of the same color.

1. **Dynamics of RNAPII loading and progression through transcriptional units (Figure 3)**

*Clustering of the RNAPII and transcriptional responses*

Out of the 4’909 genes with altered kinetics, we selected 4’651 genes with an altered synthesis rate.

For these genes, we identified clusters of genes coherent for the changes in RNAPII binding and synthesis rate (for the latter we considered only the time-points profiled for RNAPII ChIP-seq). We grouped the genes based on the untreated levels of each feature (as ranking), and their log2 ratio at each time points compared to the untreated condition. To increase the weight of the basal condition, and the changes of the synthesis rate in the clustering, we replicated them 15 and 4 times, respectively.

Genes were clustered using the R implementation of the K-means clustering algorithm (nstart=20, iter.max=40). The number of clusters that best partitioned the dataset was chosen by measuring the Akaike Information Criterion (AIC), calculated as:

*AIC = 2 * n * k + D*

where *n* is the number of items, *k* the number of clusters (we considered all clustering partitions with *n* from 2 to 40 clusters) and *D* the within-cluster sum of squares. To penalize the choice of a high number of clusters with a modest gain in clustering quality, we considered the difference (Diff) in the AIC score between the worst and the best clustering combination. We selected the number of clusters that was able to score no more than 5% of Diff worst than the best clustering combination. Using this method we chose 14 clusters as the optimal number of clusters, 2 of which were removed due to the low number of genes represented (13 and 27).

*Modeling RNAPII dynamics at each cluster*

We modeled RNAPII progression with 4 steps: (i) the flux between the nucleoplasm and promoters (p_1_; including the initiation step), (ii) the rate of release from promoter to GB (pause-release, p_2_), (iii) the rate of transition from GB to TES regions (elongation, p_3_), and (iv) the rate of transition from TES regions to the nucleoplasm (TES release, p_4_). We used the median synthesis rates (k1) and the median RNAPII signal quantified at promoter (Rtss), GB (Rgb) and TES (Rtes) regions for each cluster at each time point to infer the rates of RNAPII loading and progression in the untreated condition and following MYC-ER activation.

We modeled the variation of RNAPII ChIP-seq at different regions as the balance between the RNAPII incoming within that region and the RNAPII released. In particular, we modeled:

- the variation of RNAPII at promoters (Rtss’) as the balance between the RNAPII incoming from nucleoplasm and the RNAPII released to either gene-body or nucleoplasm
- the variation of RNAPII at gene-bodies (Rgb’) as the balance between the RNAPII entering from the promoter and the polymerase entering the TES region
- the variation of RNAPII at the TES region (Rtes’) as the balance between the polymerase entering from the gene-body and the polymerase released to the nucleoplasm

Translating this into equations:

*Rtss’ (mol/h) = Rfree (mol) * p_+1_ (1/h) - Rtss (mol) * p_-1_ (1/h) - Rtss (mol) * p_2_ (1/h)*

*Rgb’ (mol/h) = Rtss (mol) * p_2_ (1/h) - Rgb (mol) * p_3_ (1/h)*

*Rtes’ (mol/h) = Rgb (mol) * p_3_ (1/h) - Rtes (mol) * p_4_ (1/h)*

We also modeled the rate of synthesis as the product between the RNAPII at gene-bodies and the rate of elongation (Danko et al. 2013):

*k1 (mol/h) = Rgb (mol) * p_3_ (1/h)*

We simplified the modeling of the variation of RNAPII at promoters by aggregating the exchange between the nucleoplasm and the promoter into a single step, which becomes a flux (p_1_, while from here on we refer collectively to p_1-4_ as rates for simplicity). The complete model is:

*Rtss’ (mol/h) = p_1_ (mol/h) - Rtss (mol) * p_2_ (1/h)*

*Rgb’ (mol/h) = Rtss (mol) * p_2_ (1/h) - Rgb (mol) * p_3_ (1/h)*

*Rtes’ (mol/h) = Rgb (mol) * p_3_ (1/h) - Rtes (mol) * p_4_ (1/h)*

*k1 (mol/h) = Rgb (mol) * p_3_ (1/h)*

While p_2_, p_3_ and p_4_ are strictly positive kinetic rates, p_1_ is a flux that can be either positive (recruitment of polymerase flowing from the nucleoplasm to promoters) or negative (loss of polymerase flowing from promoters to the nucleoplasm). The focus of this study is on the modulation of rates induced by MYC activation and not on their absolute values. The latter would require determining a coefficient of conversion between read numbers and molecule numbers, and goes beyond the aim of this work.

The temporal profiles of p_1_, p_2_, p_3_ and p_4_ can be obtained directly by the solution of the system of equations above, by estimating Rtss’, Rgb’ and Rtes’ from the time course of Rtss, Rgb and Rtes. We fit impulse models (Chechik and Koller 2009) on the temporal profiles of p_1-4_, and we optimized the model by minimizing the error on the data calculated by the numerical solution of the system of differential equations. The full model has 24 parameters (6 parameters per each impulse model function), and is selected through the Nelder-Mead algorithm, as implemented by “optim” function of R.

Variations of p_1-4_ after MYC induction, for each cluster, is represented in Fig 3c as the log2ratio compared to the untreated condition. In order to visualize in a logarithmic scale also negative fluxes, we plotted the log2ratio of their absolute value in a scale from blue to yellow.

We estimated the importance of each rate in explaining the data by implementing reduced models (Fig 3d). These models adopted the impulse model function just for one step of the polymerase progression and modeled the other three rates as constant. These models have 9 parameters, which were obtained using the optimization procedure described above. We assessed the relative importance of each rate by their explained variance, calculated as:

*sum(( model - experiment )^2^)/sum((<experiment> - experiment)^2^)*

Modeling performance was evaluated through a 10-fold cross validation method. For each cluster we fitted the model on the 90% of the genes, and tested it on the remaining 10%. The modeling result of each feature was normalized by the ratio between the test set and the training set initial values, to avoid the bias due to different initial conditions and to focus on the error of the differential response.

1. **Dynamics of RNAPII elongation and pre-mRNA processing (Figure 4)**

*Half-response times*

Half-response times of synthesis, processing, pre-mRNA were obtained using the functional form of the kinetic rates estimated by INSPEcT. For each gene, rates and concentrations are smooth function of time, whose parameters have been estimated within the INSPEcT procedure “modelRates”. We sampled these functions at the minute resolution within the range of the MYC activation (from 0h to 16h OHT treatment), and we identified the time at which the half of the maximum response was achieved for synthesis, processing, pre-mRNA and total-mRNA.

*Modeling transcriptional dynamics with constant processing rates*

To characterize the relevance of the modulation of the processing rate on the dynamics of pre-mRNA, we evaluated how half-response times of pre-mRNA would be affected in case the processing rates were forced to remain constant at the levels of the 0h OHT untreated condition. To this purpose, we substituted the smooth function associated to the processing rate of the genes under analysis with a constant equal to the time-zero value of the original, leaving all the other rates unchanged, and calculated half-response times of the pre-mRNA as described above.

*Modeling RNAPII dynamics with constant elongation rates*

To characterize the relevance of the modulation of the RNAPII elongation rate on the RNAPII dynamics, we evaluated how the latter are affected by an elongation rate forced to remain constant at the levels of the 0h OHT untreated condition. We substituted the smooth function associated to the elongation rate of cluster 3 with a constant equal to the time-zero value of the original, leaving all the other rates unchanged.

*Analysis of splicing alterations*

We searched for splicing alterations using the DEXseq (Anders et al. 2012) Bioconductor package, which was developed to test for differential exon usage in comparative RNA-seq experiments. We applied this method to both exons and introns, in order to identify events of *differential exon inclusion* or *differential intron retention*. First of all, we defined the set exons and introns as described in the section “Quantification of raw read counts” in the Section 4b of this document. Total RNA reads were assigned and quantified at matching exons. Unassigned reads were then assigned and quantified at matching introns. DEXseq was applied to compare read counts jointly in exons and introns of each gene for each time point against the untreated condition. We considered as significant events with an adjusted p-value lower than 0.05. To increase the likelihood of identifying splicing defects while avoiding events related to transcriptional modulation, we excluded genes with more than one intron or exon with differential usage per time point, thus focusing only on transcriptional units affect at the level of single exons or introns. In addition, we discarded the events occurring in the first two introns from the 5’ end of genes.

1. **References**

Anders S, Reyes A, Huber W. 2012. Detecting differential usage of exons from RNA-seq data. **22**: 2008–2017.

Chechik G, Koller D. 2009. Timing of gene expression responses to environmental changes. *Journal of Computational Biology* **16**: 279–290.

Danko CG, Hah N, Luo X, Martins AL, Core L, Lis JT, Siepel A, Kraus WL. 2013. Signaling Pathways Differentially Affect RNA Polymerase II Initiation, Pausing, and Elongation Rate in Cells. *Molecular Cell* **50**: 212–222.

de Pretis S, Kress T, Morelli MJ, Melloni GEM, Riva L, Amati B, Pelizzola M. 2015. INSPEcT: a computational tool to infer mRNA synthesis, processing and degradation dynamics from RNA- and 4sU-seq time course experiments. **31**: 2829–2835.

Feng J, Liu T, Qin B, Zhang Y, Liu XS. 2012. Identifying ChIP-seq enrichment using MACS. *Nat Protoc* **7**: 1728–1740.

Risso D, Ngai J, Speed TP, Dudoit S. 2014. Normalization of RNA-seq data using factor analysis of control genes or samples. *Nat Biotechnol* **32**: 896–902.

Roberts A, Goff L, Pertea G, Kim D, Kelley DR, Pimentel H, Salzberg SL, Rinn JL, Pachter L, Trapnell C. 2012. Differential gene and transcript expression analysis of RNA-seq experiments with TopHat and Cufflinks. *Nat Protoc* **7**: 562–578.

Sabò A, Kress TR, Pelizzola M, de Pretis S, Gorski MM, Tesi A, Morelli MJ, Bora P, Doni M, Verrecchia A, et al. 2014. Selective transcriptional regulation by Myc in cellular growth control and lymphomagenesis. *Nature* **511**: 488–492.

Smale DBAP-JA-JTIBMLGNDBS, Pandya-Jones A, Tong A-J, Barozzi I, Lissner MM, Natoli G, Black DL, Smale ST. 2012. Transcript Dynamics of Proinflammatory Genes Revealed by Sequence Analysis of Subcellular RNA Fractions. *CELL* **150**: 279–290.

Walz S, Lorenzin F, Morton J, Wiese KE, Eyss von B, Herold S, Rycak L, Dumay-Odelot H, Karim S, Bartkuhn M, et al. 2014. Activation and repression by oncogenic MYC shape tumour-specific gene expression profiles. *Nature* 1–17.

Zhang Y, Liu T, Meyer CA, Eeckhoute J, Johnson DS, Bernstein BE, Nusbaum C, Myers RM, Brown M, Li W, et al. 2008. Model-based analysis of ChIP-Seq (MACS). *Genome Biology* **9**: R137.
