# Supplementary material for: Integrative analysis of RNA polymerase II and transcriptional dynamics upon MYC activation
Source: Genome Res. 2017 Oct;27(10):1658–64. doi: 10.1101/gr.226035.117 (PMC5630029; doi:10.1101/gr.226035.117)
Supplement: Supplemental Material [file supp_gr.226035.117_Supplemental_Code.zip › dePretis2017_GR_code/figures/S4E.pdf]

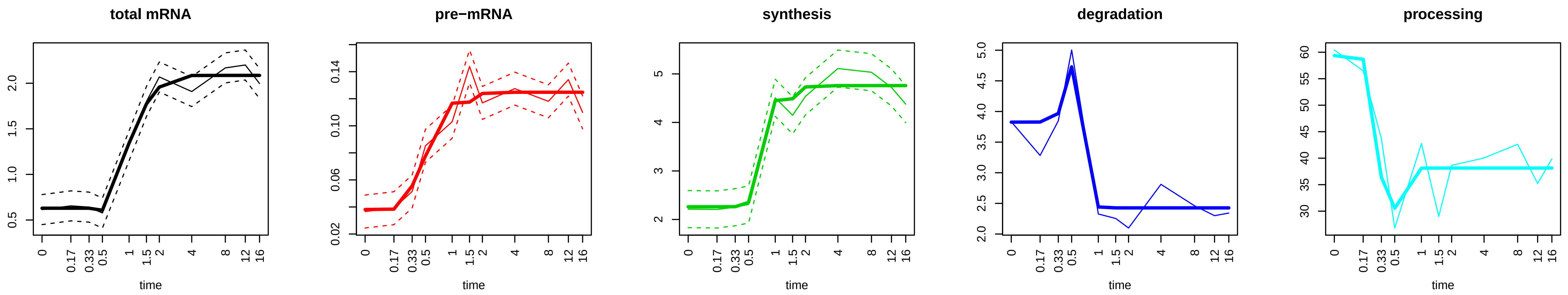

total mRNA
pre−mRNA
synthesis
degradation
processing
time
time
time
time
time
0
0.17
0.33
0.5
1
1.5
2
4
8
12
16

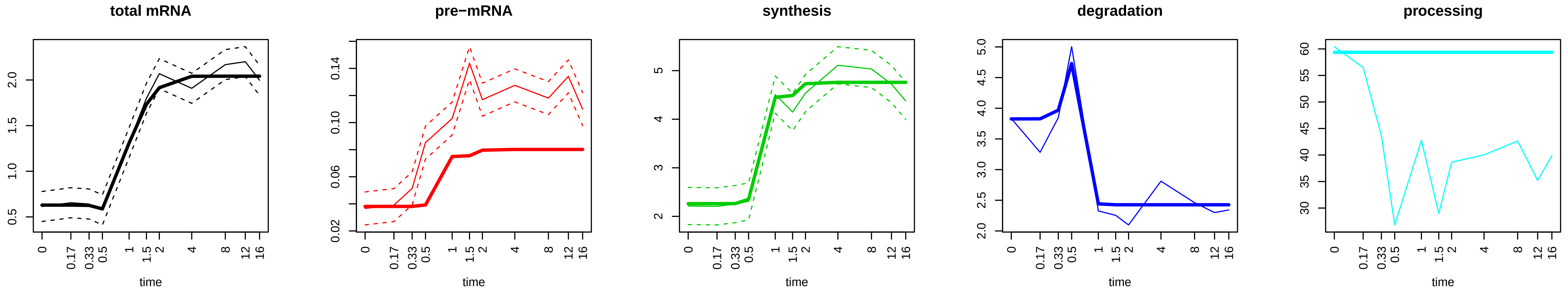

total mRNA
pre−mRNA
synthesis
degradation
processing
time
time
time
time
time
