# Supplementary figures and images for: Integrative analysis of RNA polymerase II and transcriptional dynamics upon MYC activation
Source: Genome Res. 2017 Oct;27(10):1658–64. doi: 10.1101/gr.226035.117 (PMC5630029; doi:10.1101/gr.226035.117)

**3T9MycER**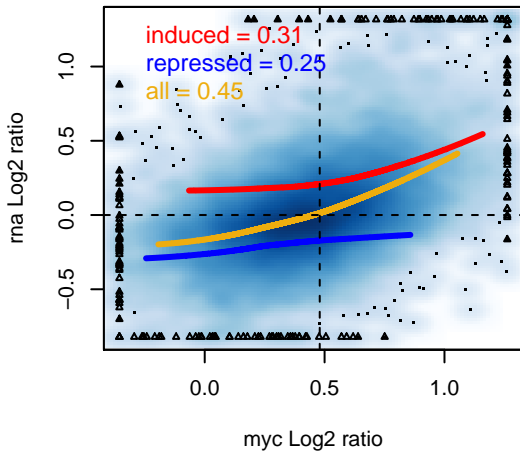**U2OSTet-Myc**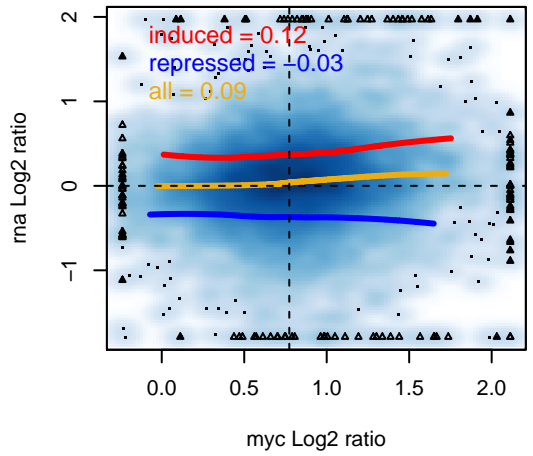**p493-6**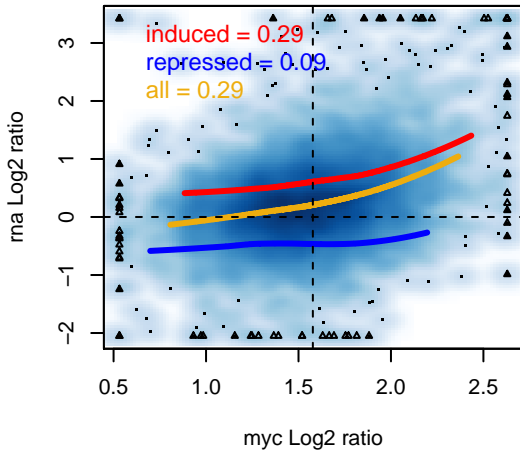**Eu-myc**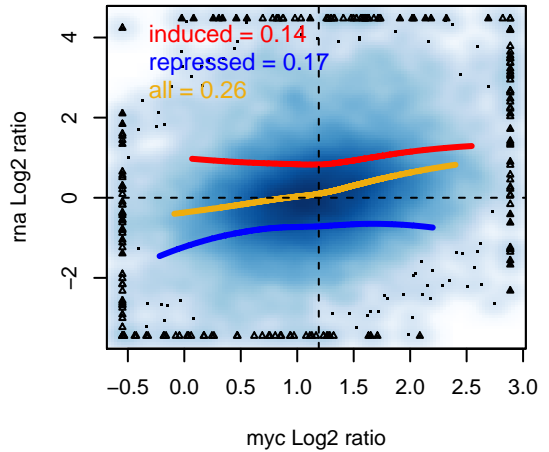**LiverTet-Myc**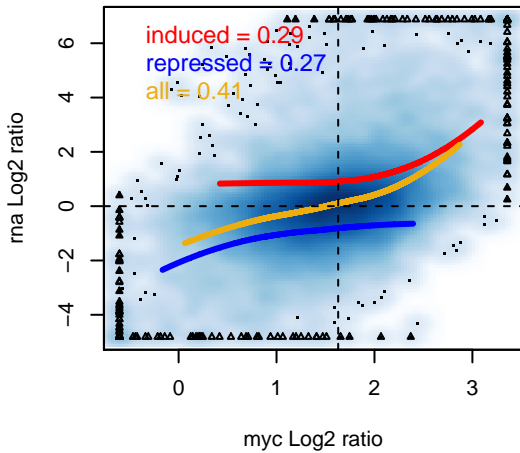

Supplement: Supplemental Material [file supp_gr.226035.117_Supplemental_Code.zip › dePretis2017_GR_code/figures/1A.pdf]

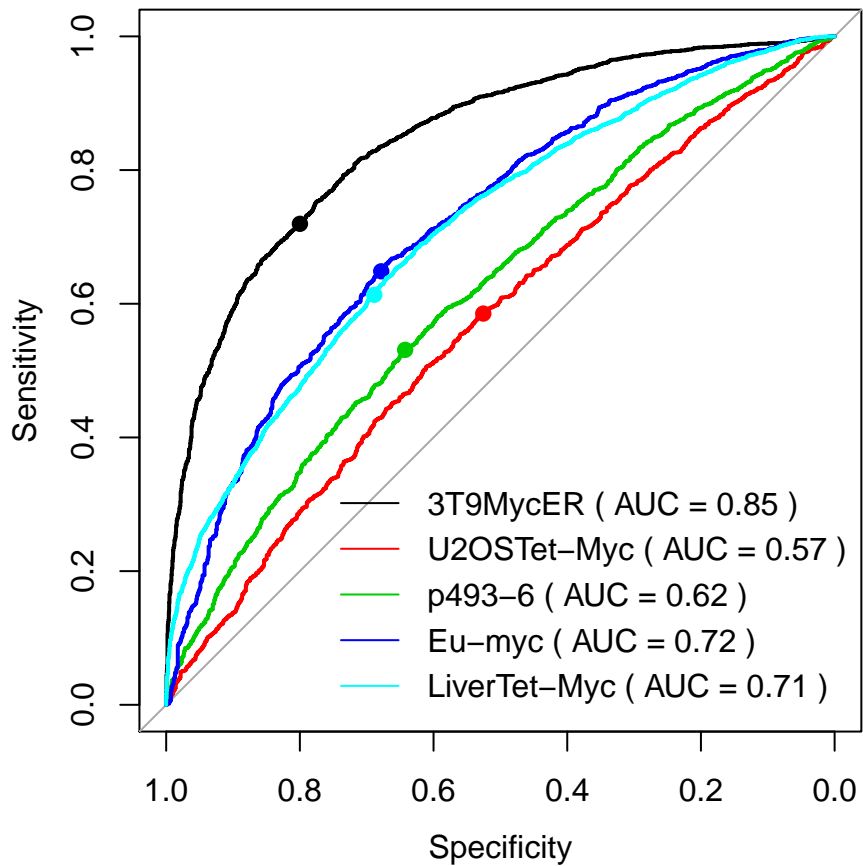

Supplement: Supplemental Material [file supp_gr.226035.117_Supplemental_Code.zip › dePretis2017_GR_code/figures/1C.pdf]

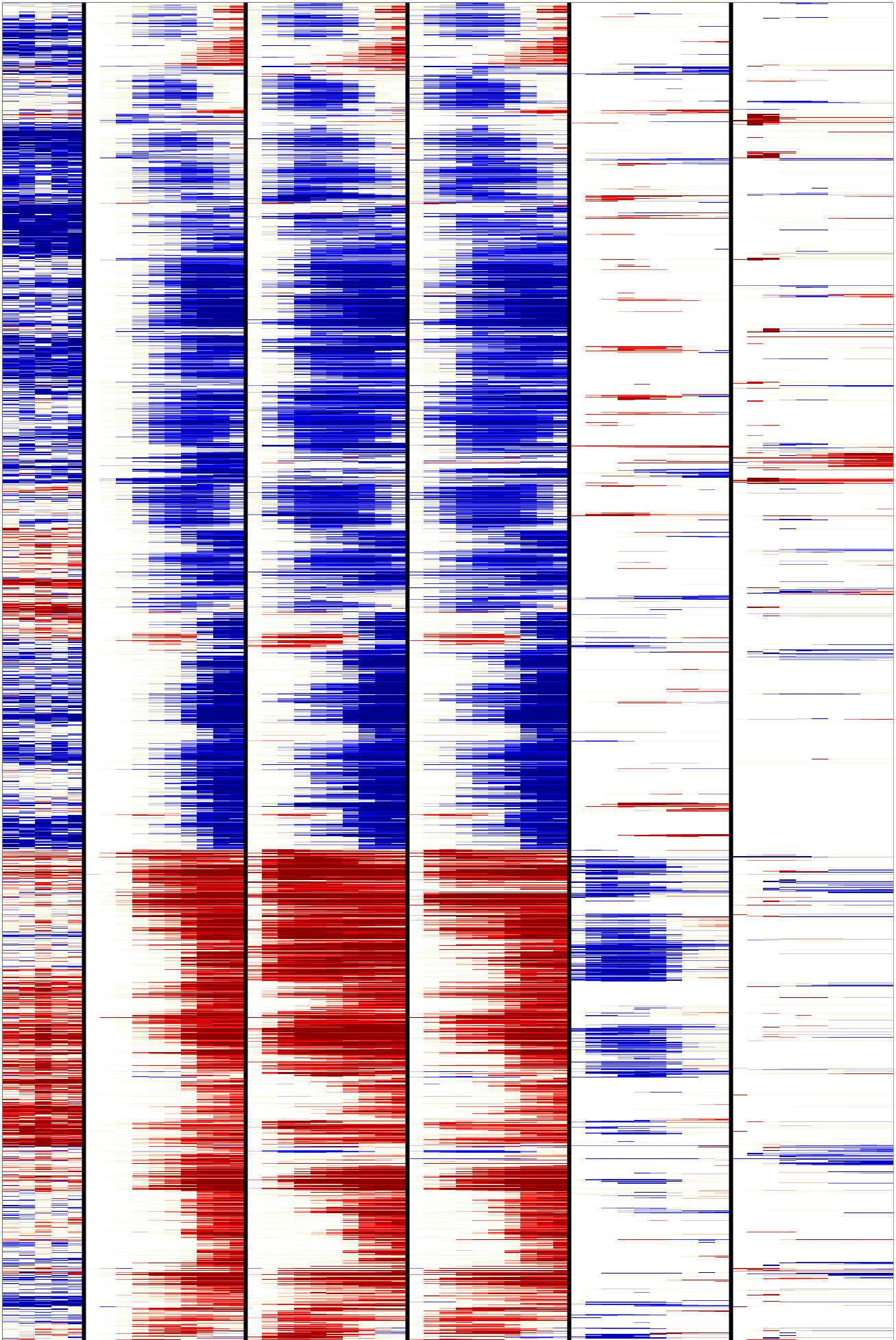

Supplement: Supplemental Material [file supp_gr.226035.117_Supplemental_Code.zip › dePretis2017_GR_code/figures/2C.pdf]

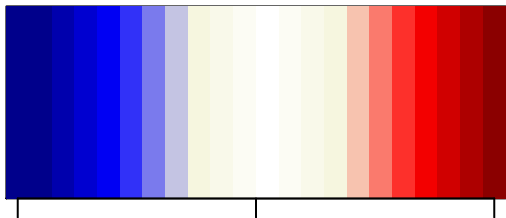

-0.58

0

0.58

Supplement: Supplemental Material [file supp_gr.226035.117_Supplemental_Code.zip › dePretis2017_GR_code/figures/2C_colscale.pdf]

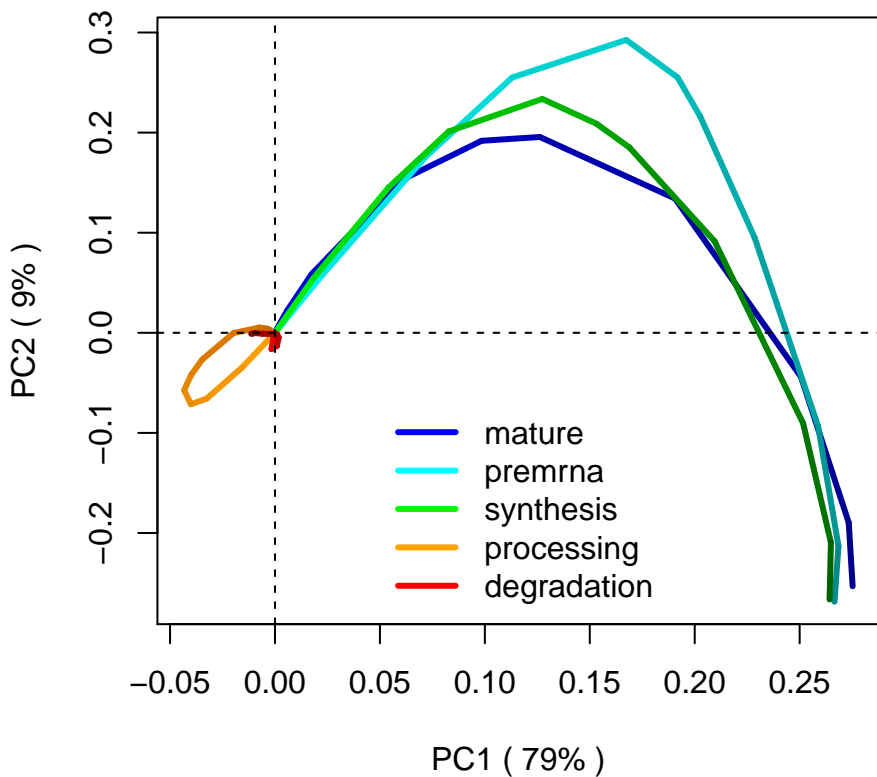

Supplement: Supplemental Material [file supp_gr.226035.117_Supplemental_Code.zip › dePretis2017_GR_code/figures/2D.pdf]

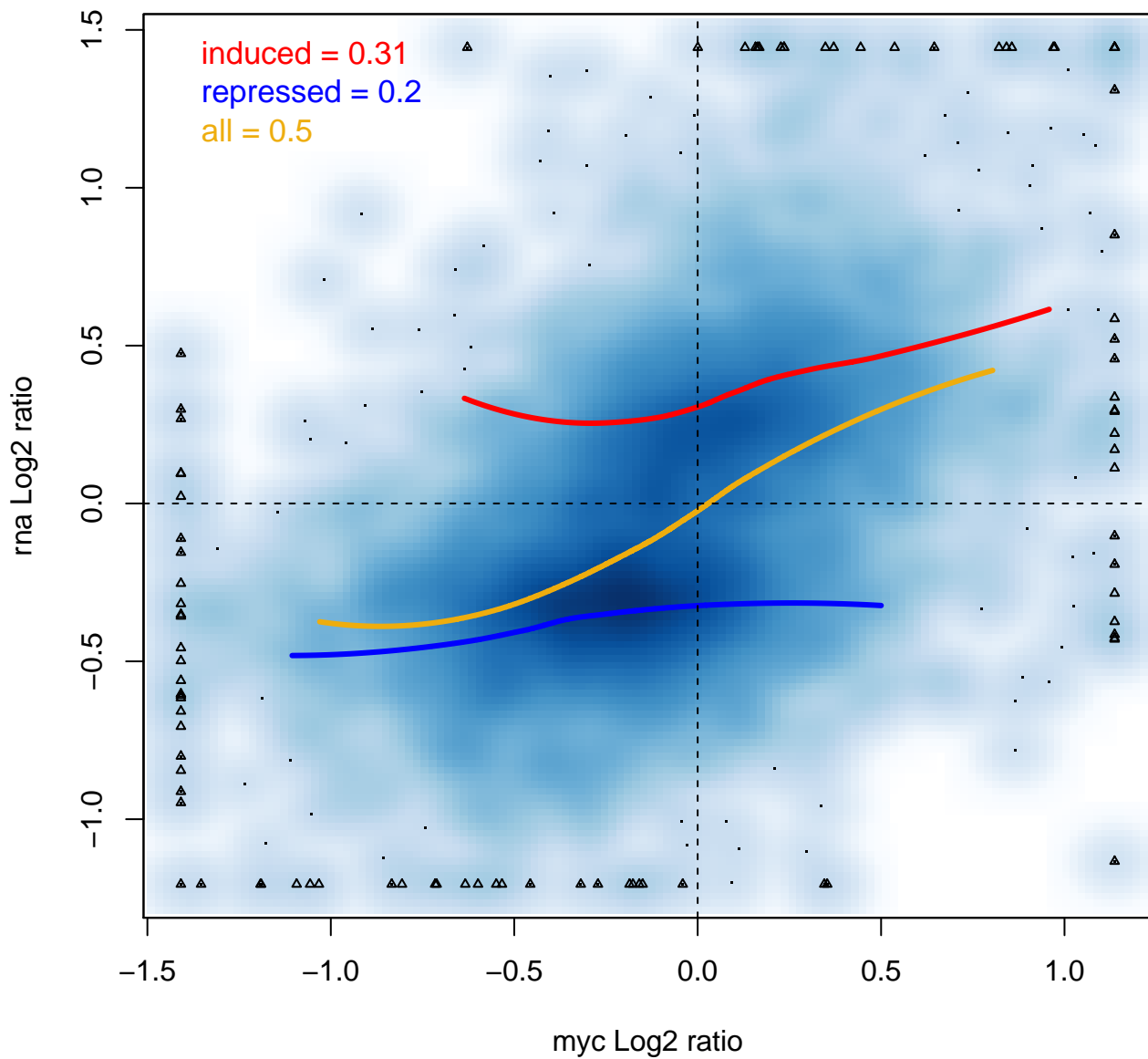

Supplement: Supplemental Material [file supp_gr.226035.117_Supplemental_Code.zip › dePretis2017_GR_code/figures/2E.pdf]

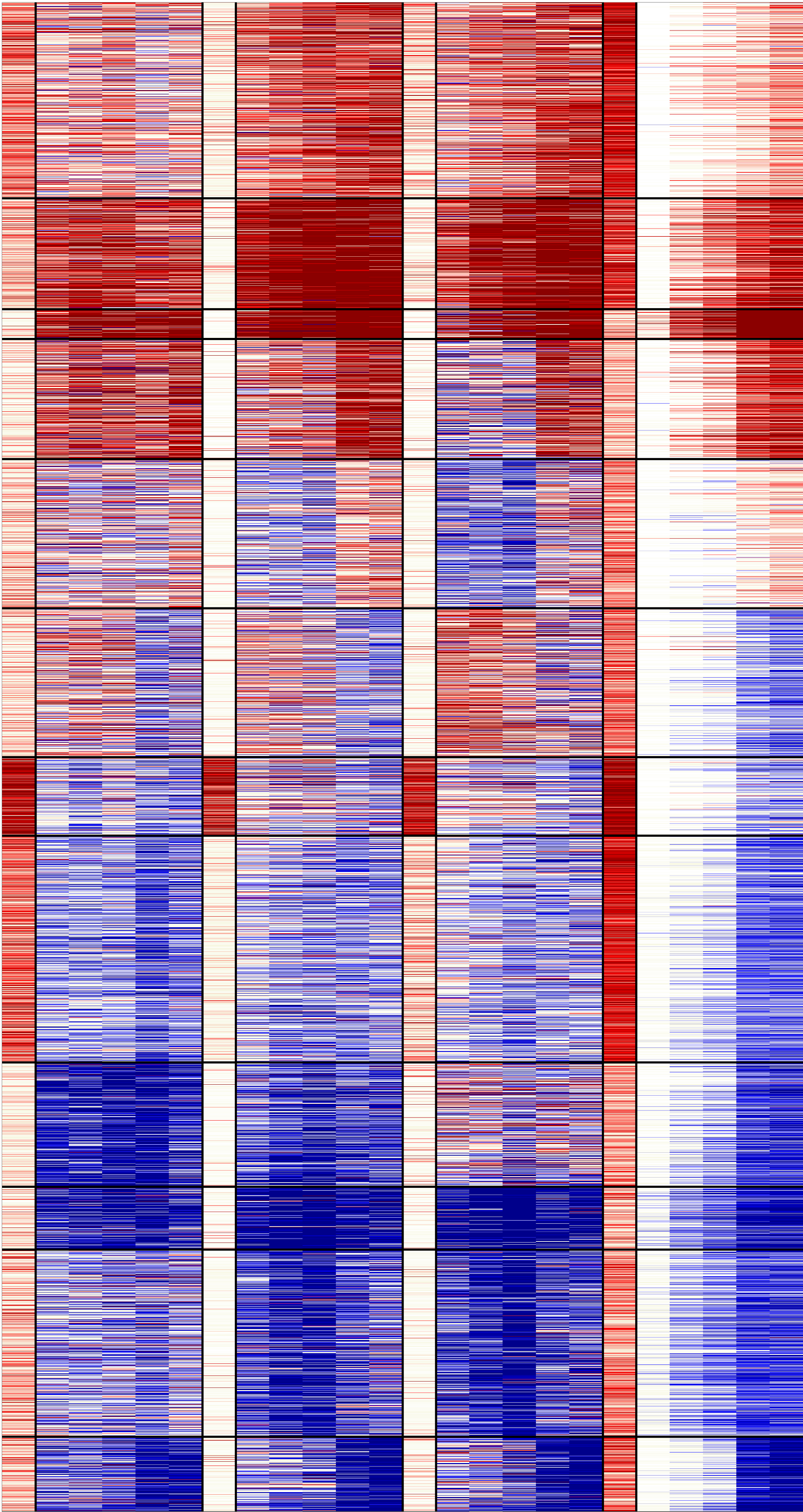

Supplement: Supplemental Material [file supp_gr.226035.117_Supplemental_Code.zip › dePretis2017_GR_code/figures/3A.pdf]

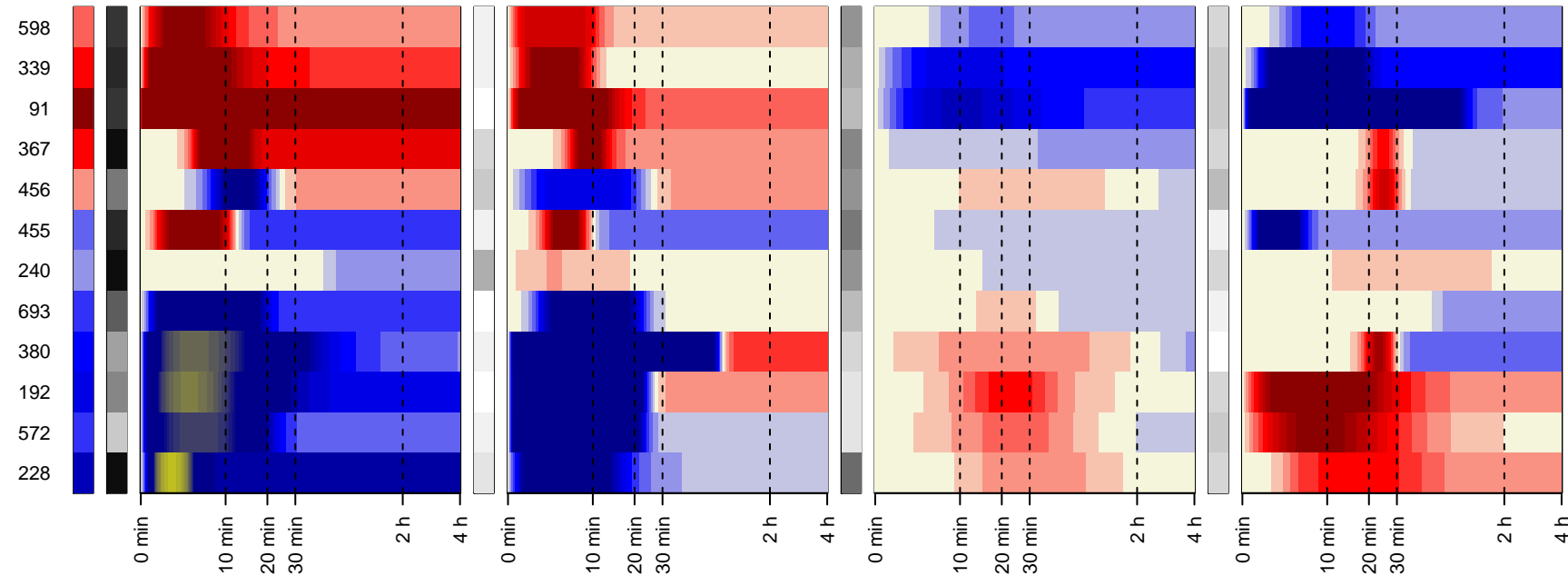

Supplement: Supplemental Material [file supp_gr.226035.117_Supplemental_Code.zip › dePretis2017_GR_code/figures/3C.pdf]

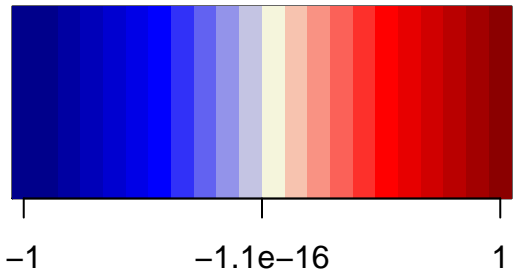

Supplement: Supplemental Material [file supp_gr.226035.117_Supplemental_Code.zip › dePretis2017_GR_code/figures/3C_colscale1.pdf]

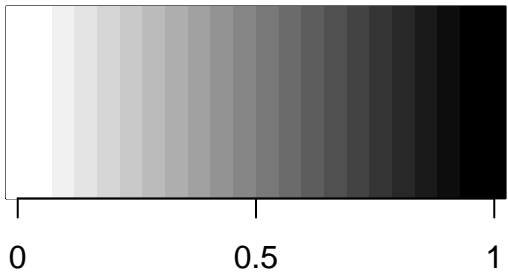

Supplement: Supplemental Material [file supp_gr.226035.117_Supplemental_Code.zip › dePretis2017_GR_code/figures/3C_colscale2.pdf]

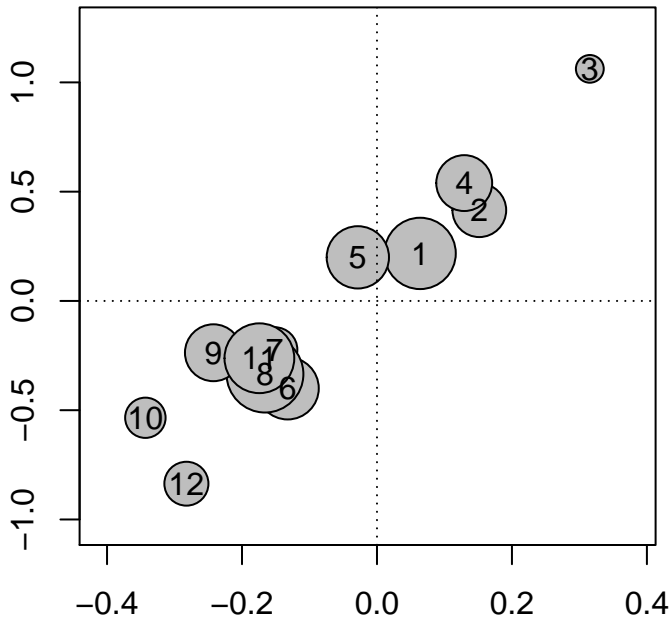

Supplement: Supplemental Material [file supp_gr.226035.117_Supplemental_Code.zip › dePretis2017_GR_code/figures/3E.pdf]

Myc Binding at 0h

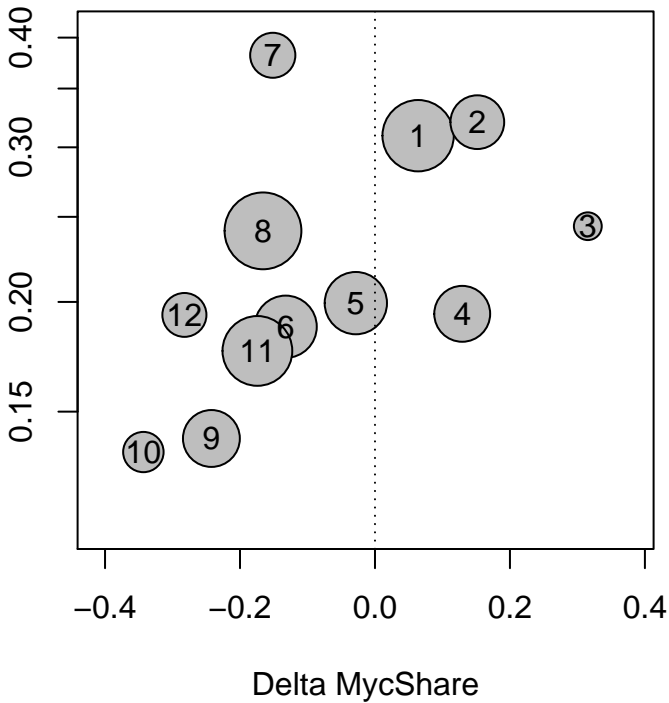

Supplement: Supplemental Material [file supp_gr.226035.117_Supplemental_Code.zip › dePretis2017_GR_code/figures/3Ebis.pdf]

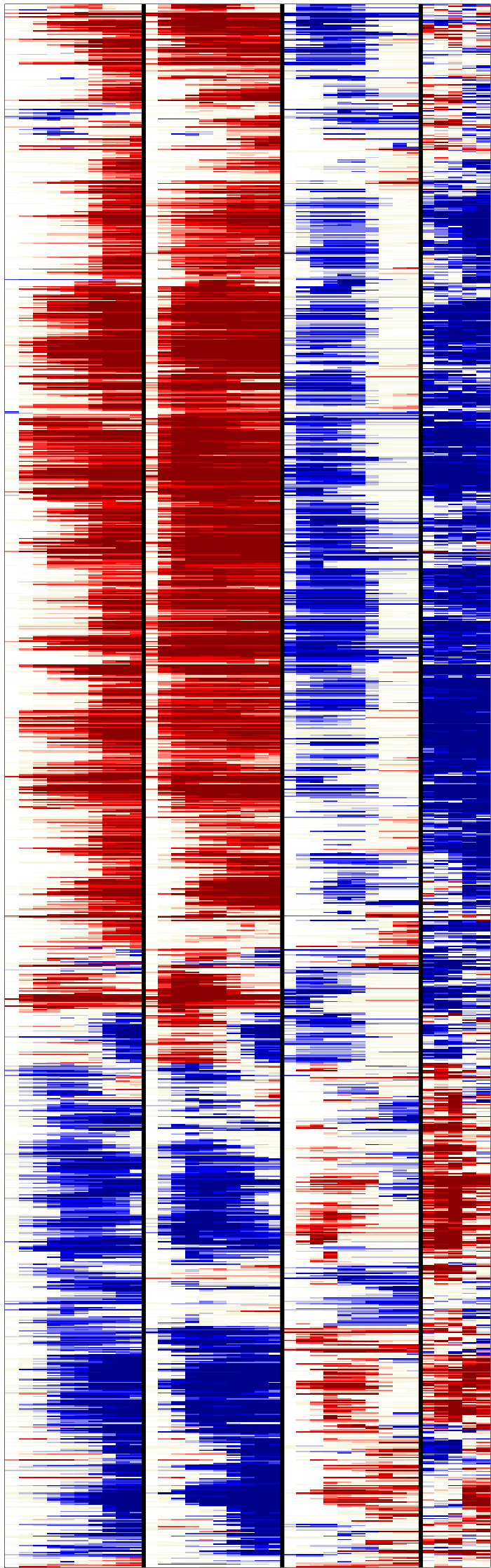

Supplement: Supplemental Material [file supp_gr.226035.117_Supplemental_Code.zip › dePretis2017_GR_code/figures/4.pdf]

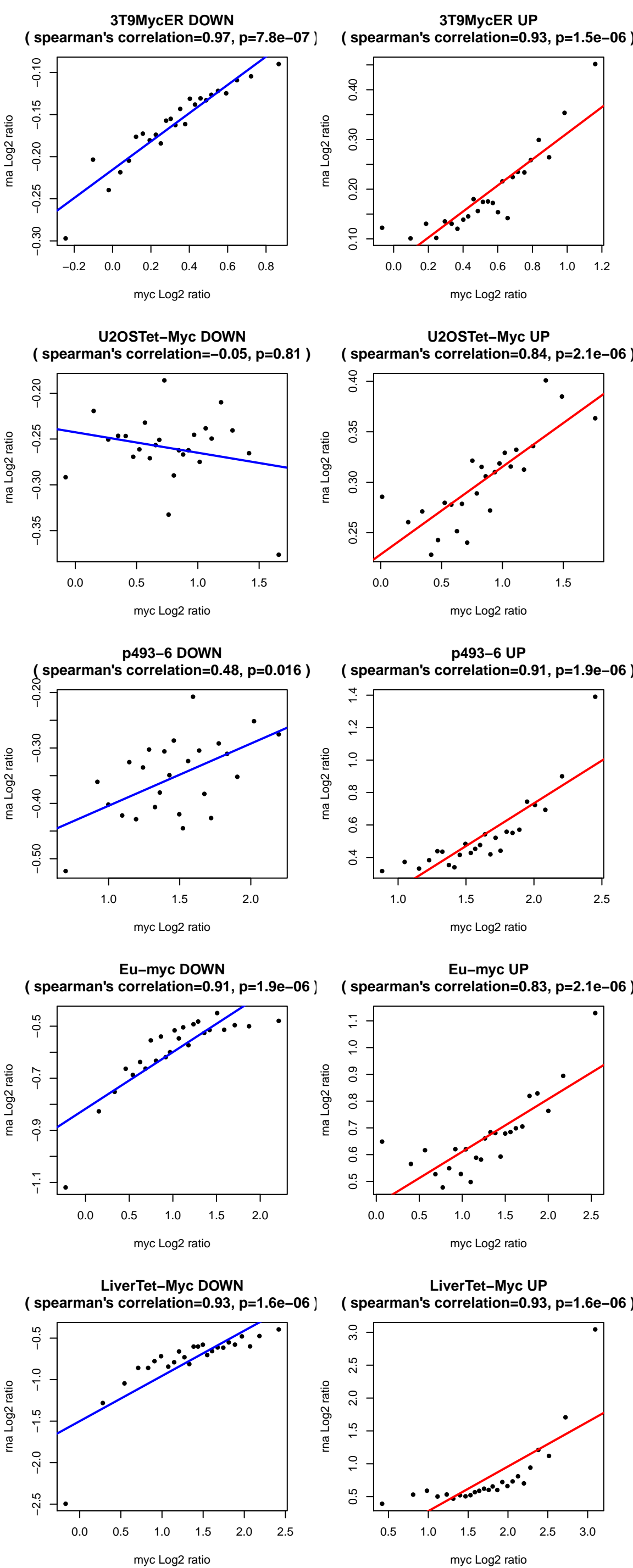

Supplement: Supplemental Material [file supp_gr.226035.117_Supplemental_Code.zip › dePretis2017_GR_code/figures/S1A.pdf]

LiverTet-Myc

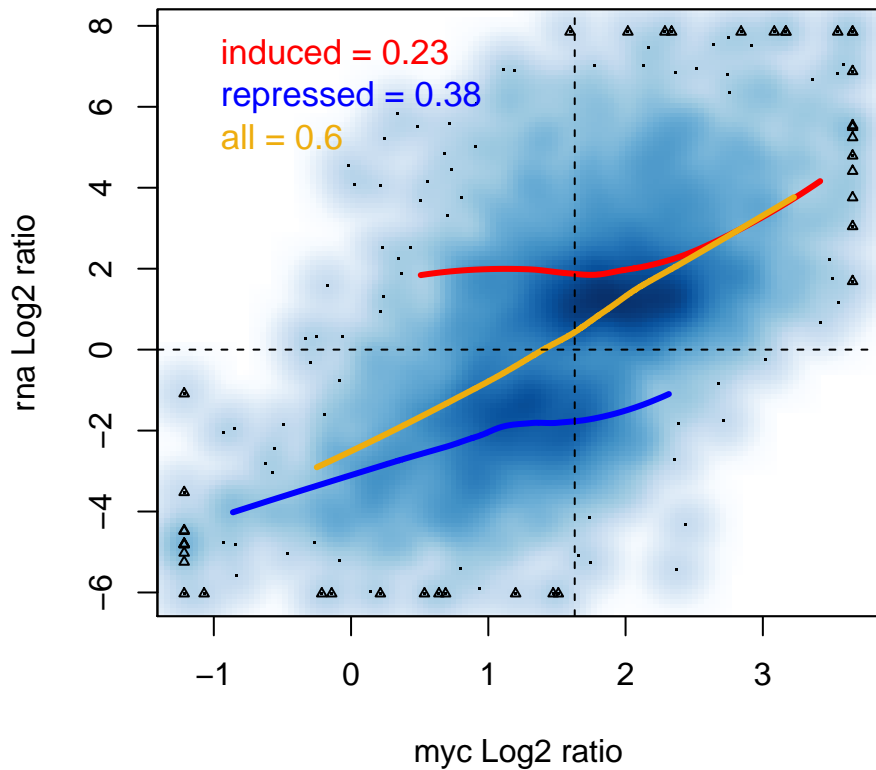

LiverTet-Myc

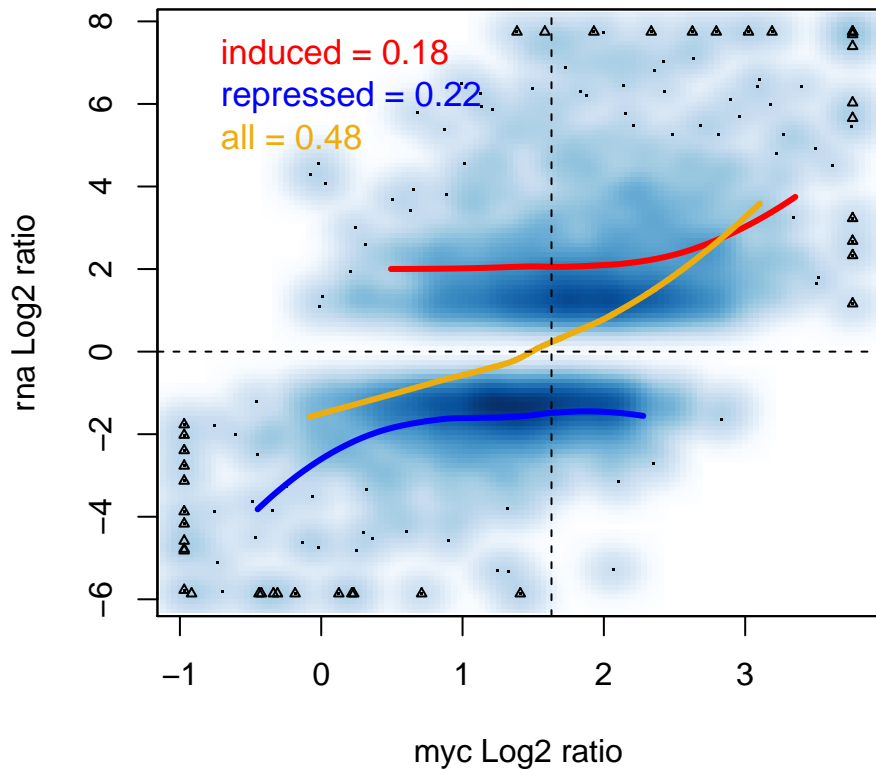

Supplement: Supplemental Material [file supp_gr.226035.117_Supplemental_Code.zip › dePretis2017_GR_code/figures/S1B.pdf]

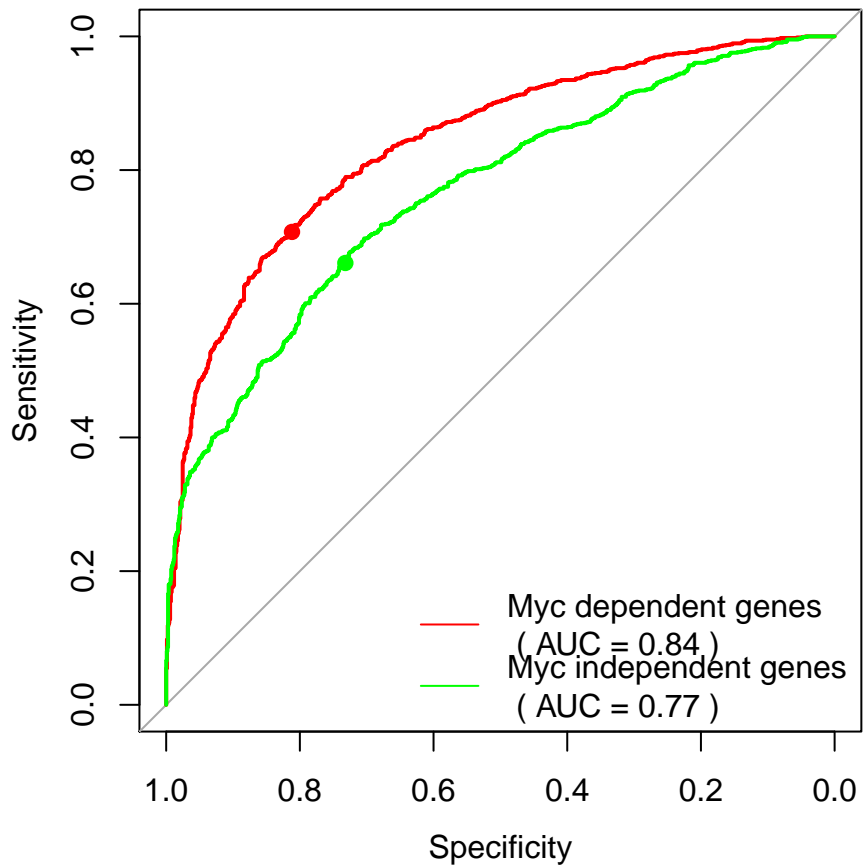

Supplement: Supplemental Material [file supp_gr.226035.117_Supplemental_Code.zip › dePretis2017_GR_code/figures/S1C.pdf]

**3T9MycER**

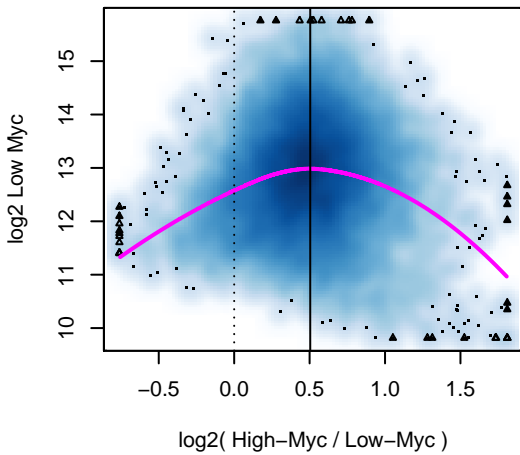

**U2OSTet-Myc**

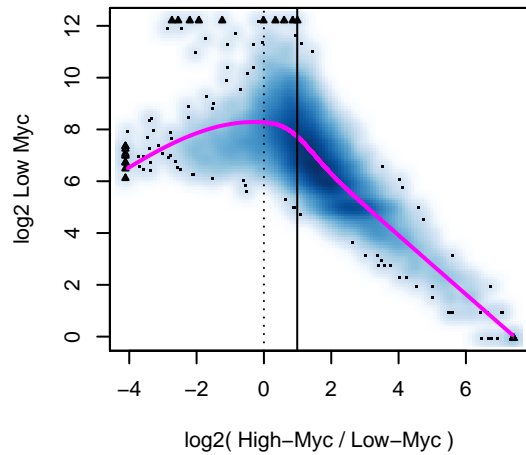

**p493-6**

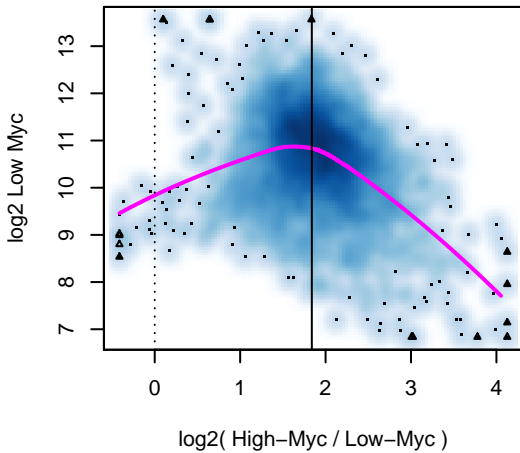

**Eu-myc**

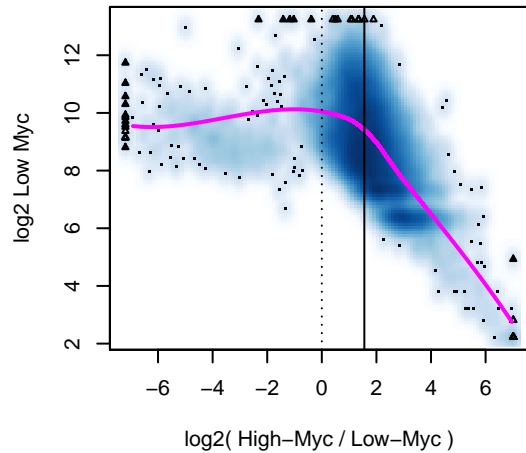

**LiverTet-Myc**

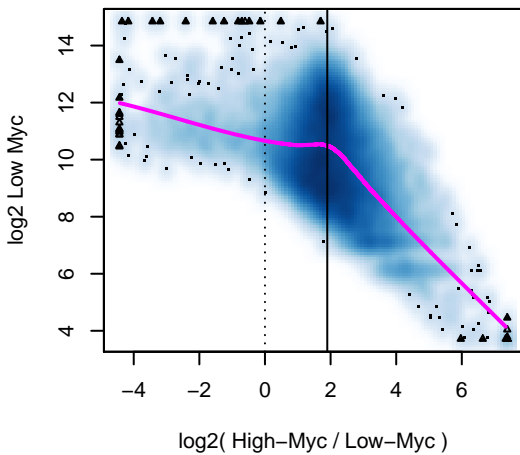

Supplement: Supplemental Material [file supp_gr.226035.117_Supplemental_Code.zip › dePretis2017_GR_code/figures/S1D.pdf]

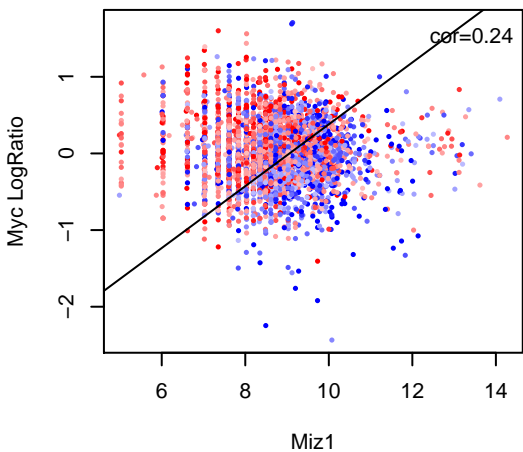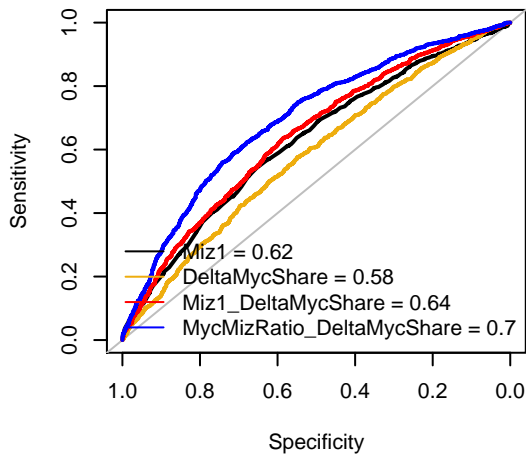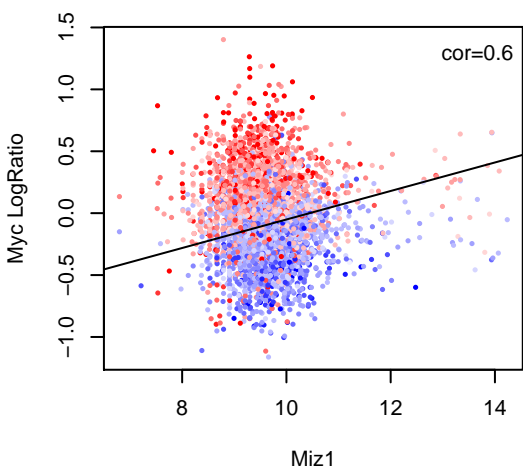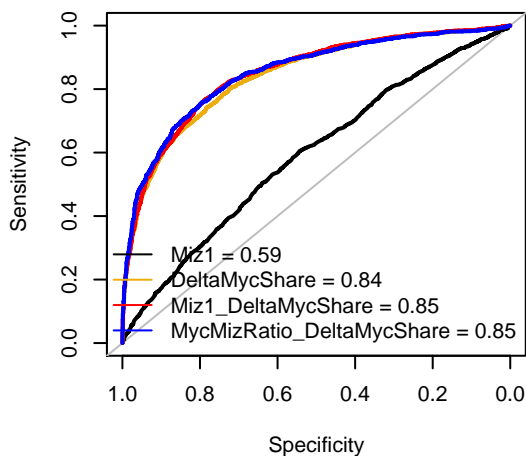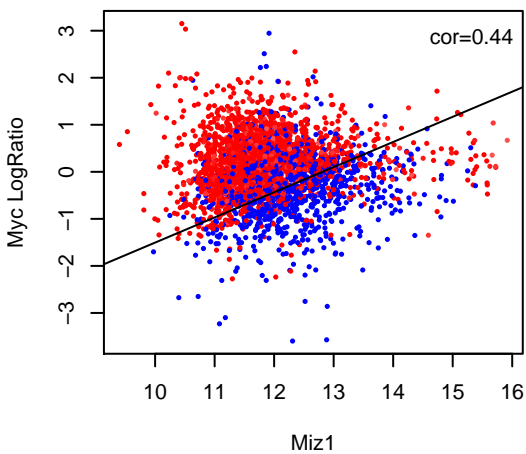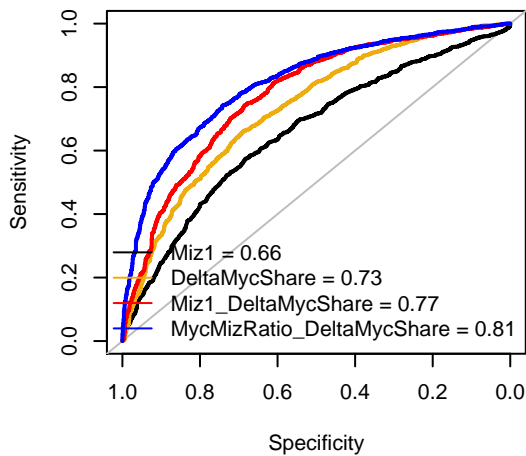

Supplement: Supplemental Material [file supp_gr.226035.117_Supplemental_Code.zip › dePretis2017_GR_code/figures/S1EF.pdf]

Density

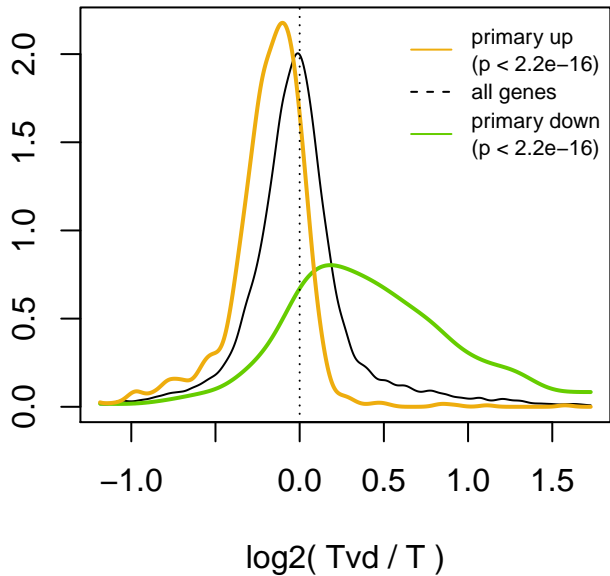

Supplement: Supplemental Material [file supp_gr.226035.117_Supplemental_Code.zip › dePretis2017_GR_code/figures/S1G.pdf]

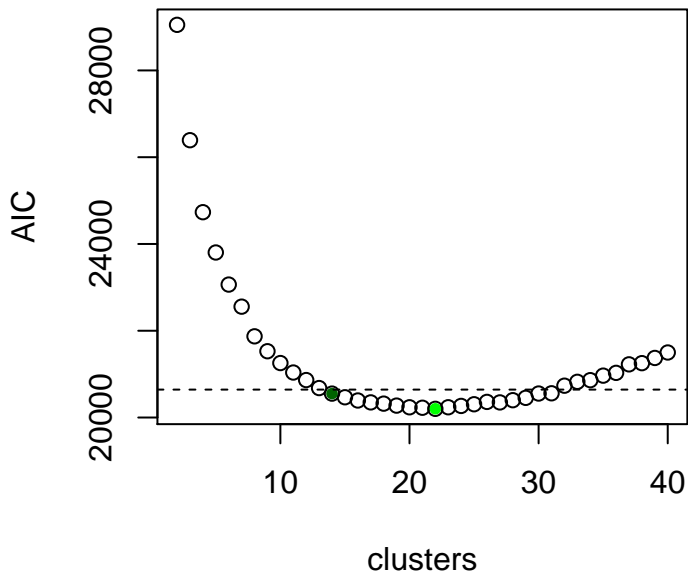

Supplement: Supplemental Material [file supp_gr.226035.117_Supplemental_Code.zip › dePretis2017_GR_code/figures/S3A.pdf]

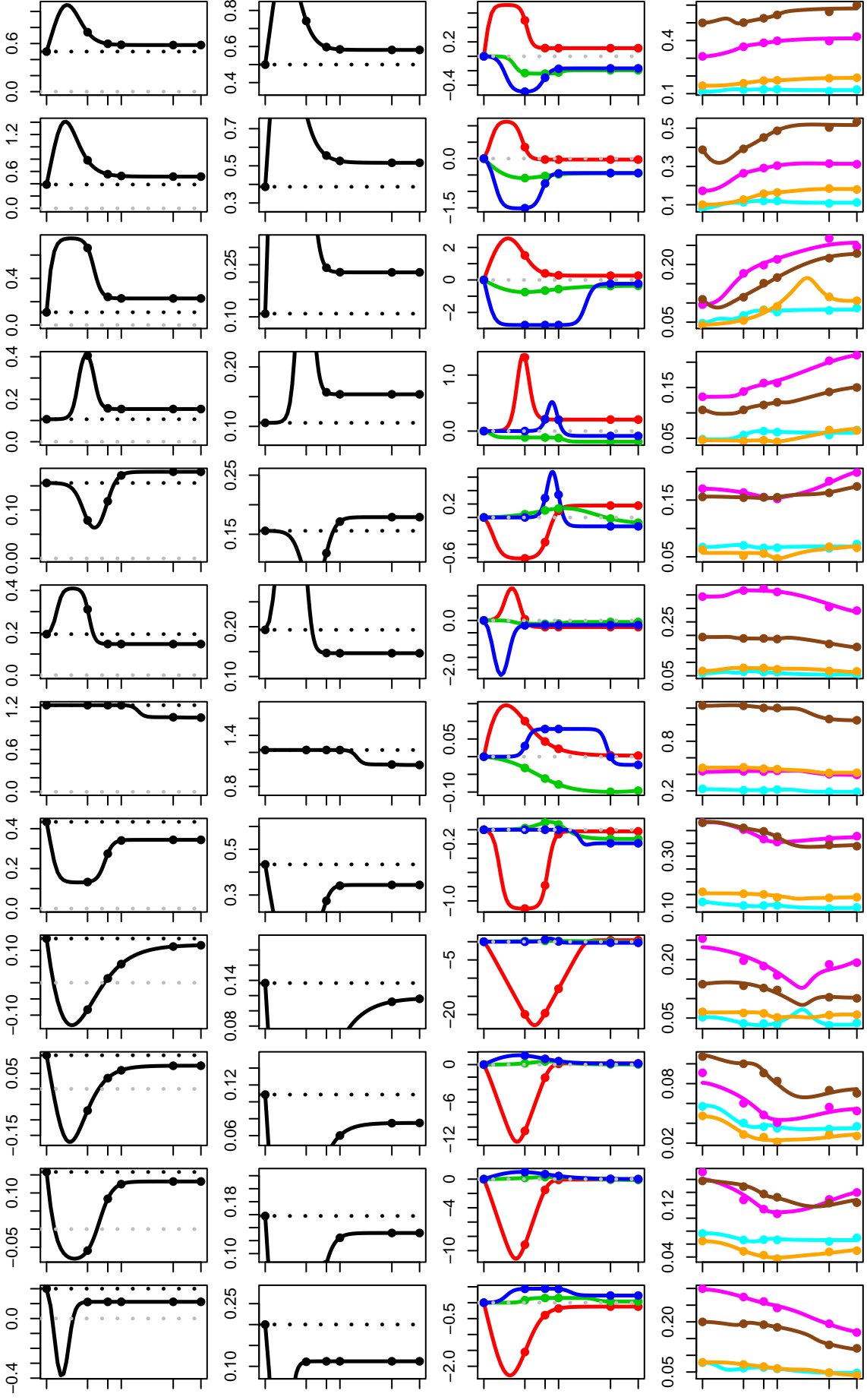

Supplement: Supplemental Material [file supp_gr.226035.117_Supplemental_Code.zip › dePretis2017_GR_code/figures/S3BC.pdf]

Fraction of explained variance

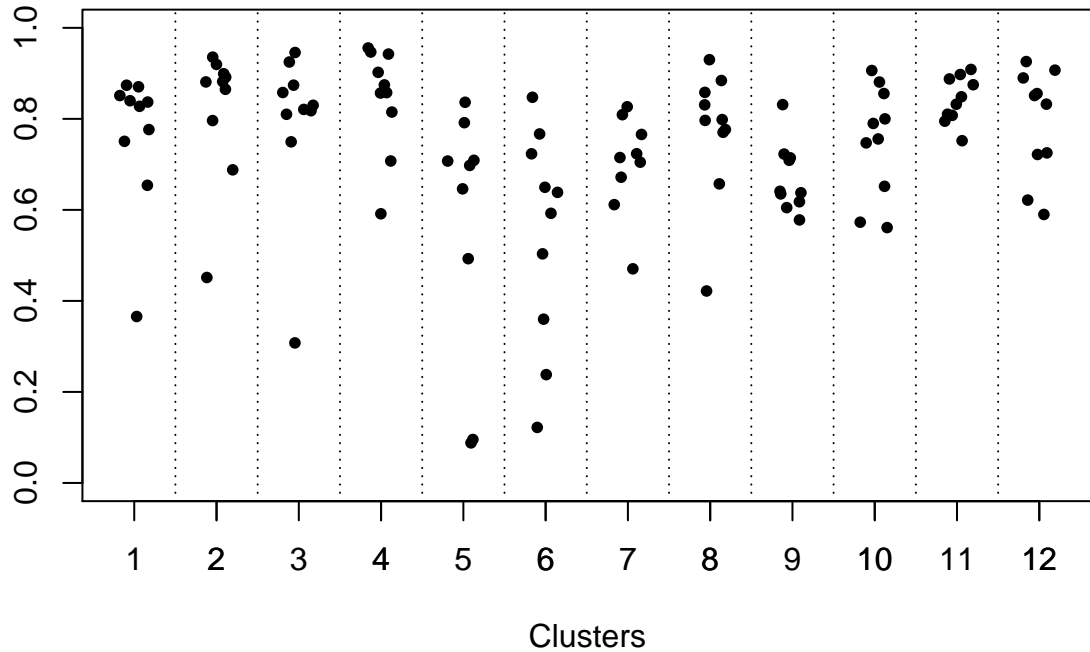

Supplement: Supplemental Material [file supp_gr.226035.117_Supplemental_Code.zip › dePretis2017_GR_code/figures/S3D.pdf]

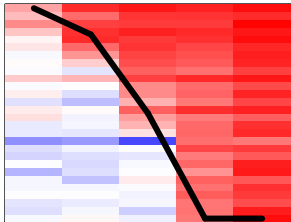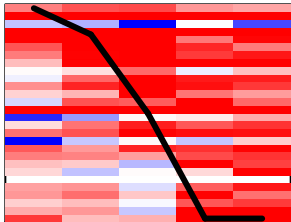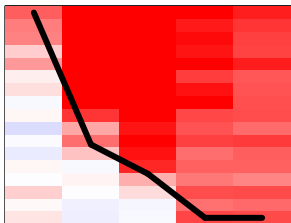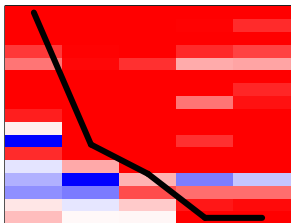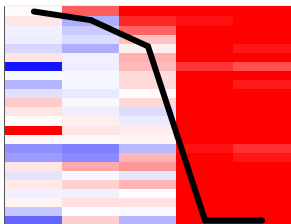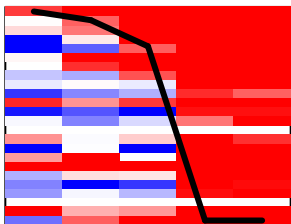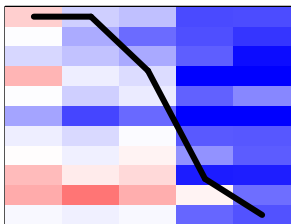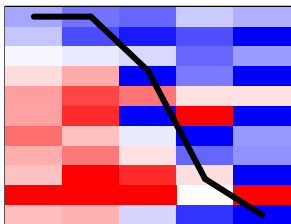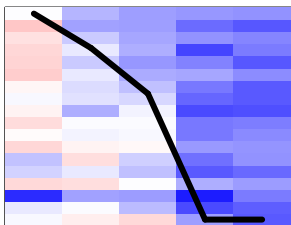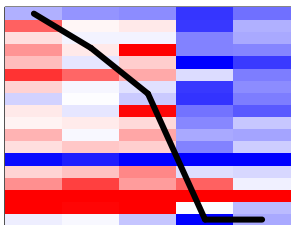

Supplement: Supplemental Material [file supp_gr.226035.117_Supplemental_Code.zip › dePretis2017_GR_code/figures/S4A.pdf]

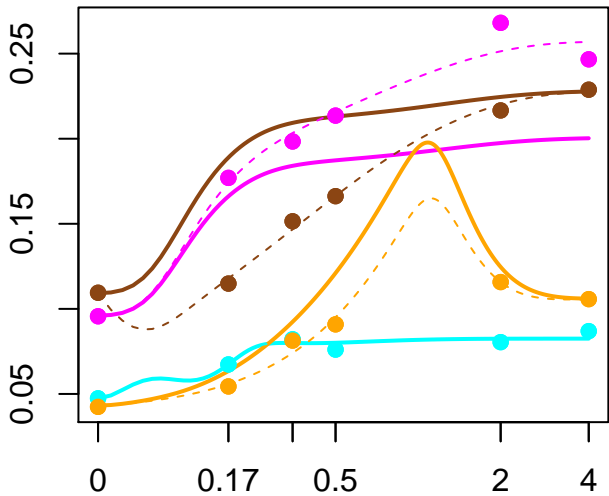

Supplement: Supplemental Material [file supp_gr.226035.117_Supplemental_Code.zip › dePretis2017_GR_code/figures/S4B.pdf]

## half-response time

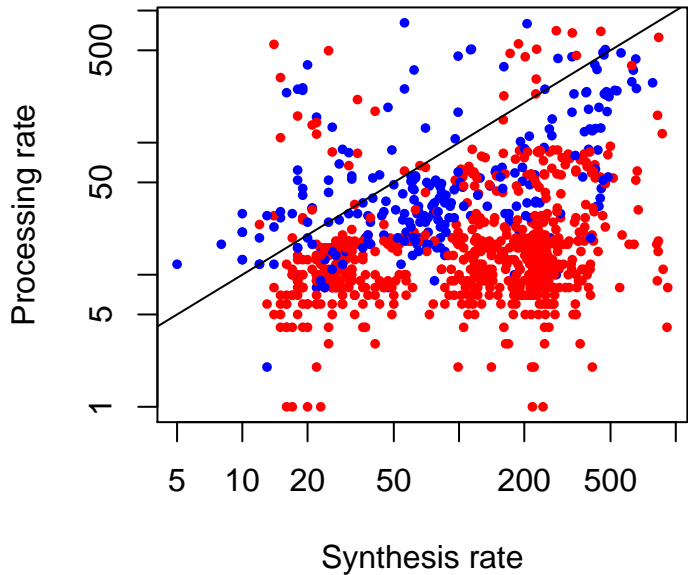

Supplement: Supplemental Material [file supp_gr.226035.117_Supplemental_Code.zip › dePretis2017_GR_code/figures/S4C.pdf]

pre-mRNA half response time (constant k2)

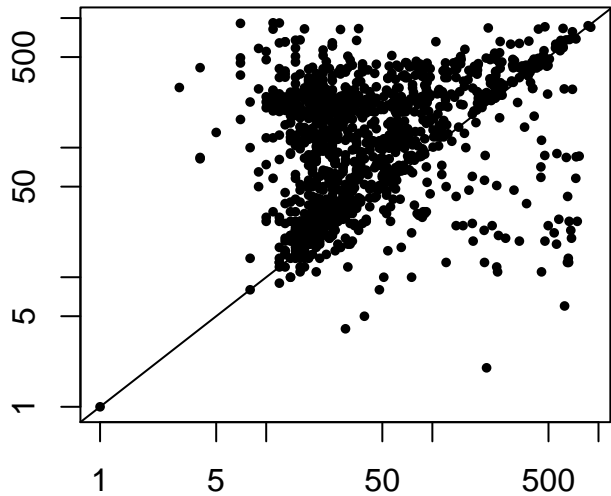

pre-mRNA half response time

Supplement: Supplemental Material [file supp_gr.226035.117_Supplemental_Code.zip › dePretis2017_GR_code/figures/S4D.pdf]

# 319481 –

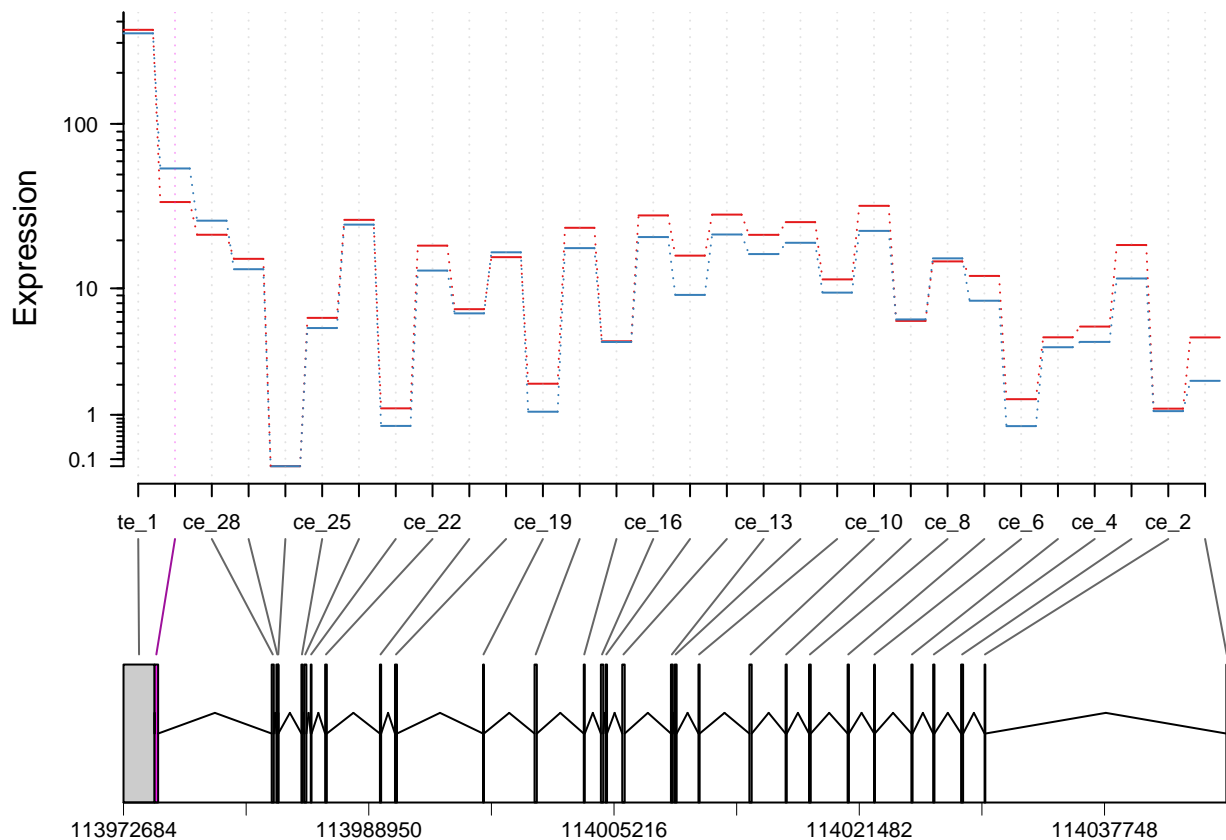

Supplement: Supplemental Material [file supp_gr.226035.117_Supplemental_Code.zip › dePretis2017_GR_code/figures/S4F.pdf]

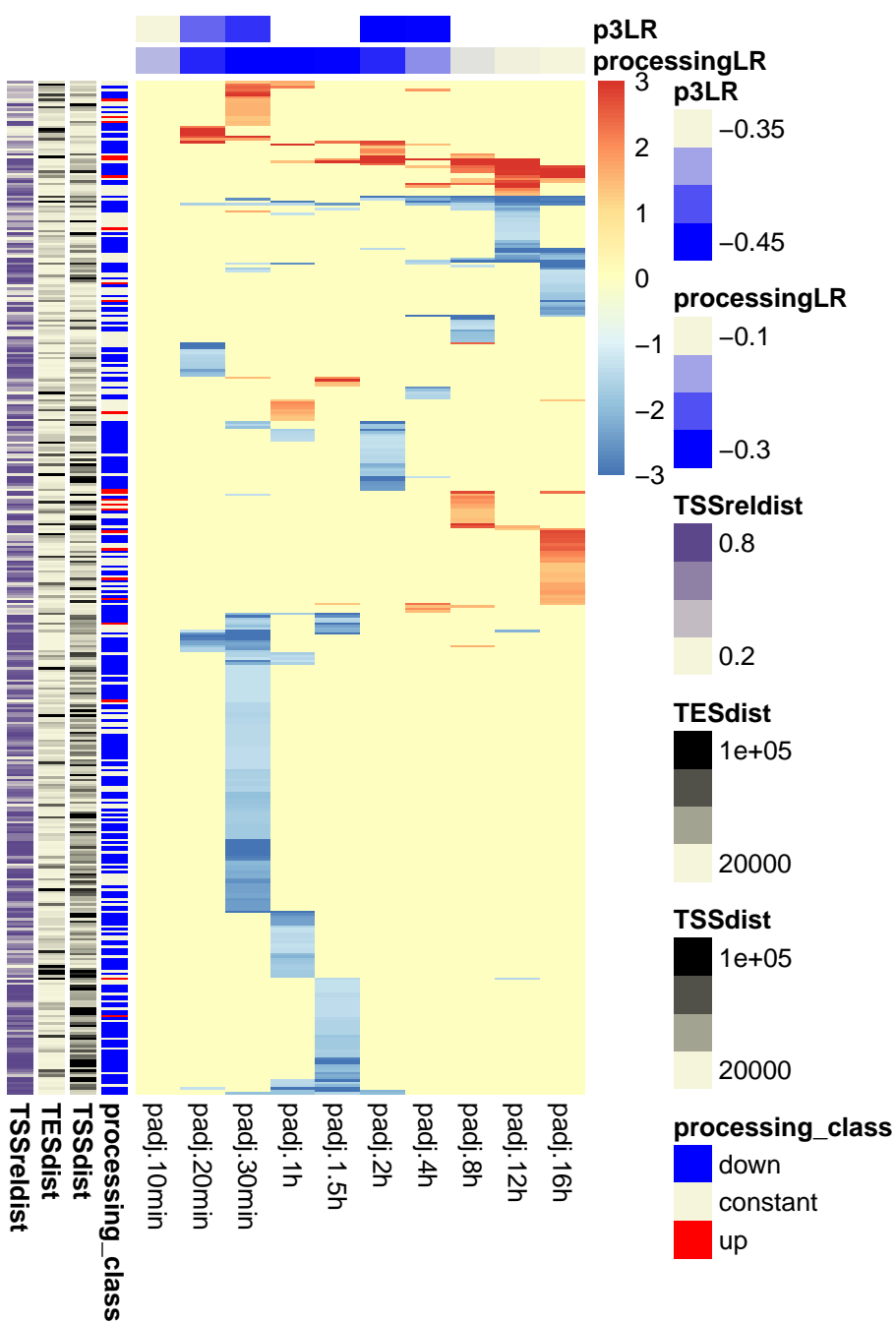

Supplement: Supplemental Material [file supp_gr.226035.117_Supplemental_Code.zip › dePretis2017_GR_code/figures/S4Ga.pdf]

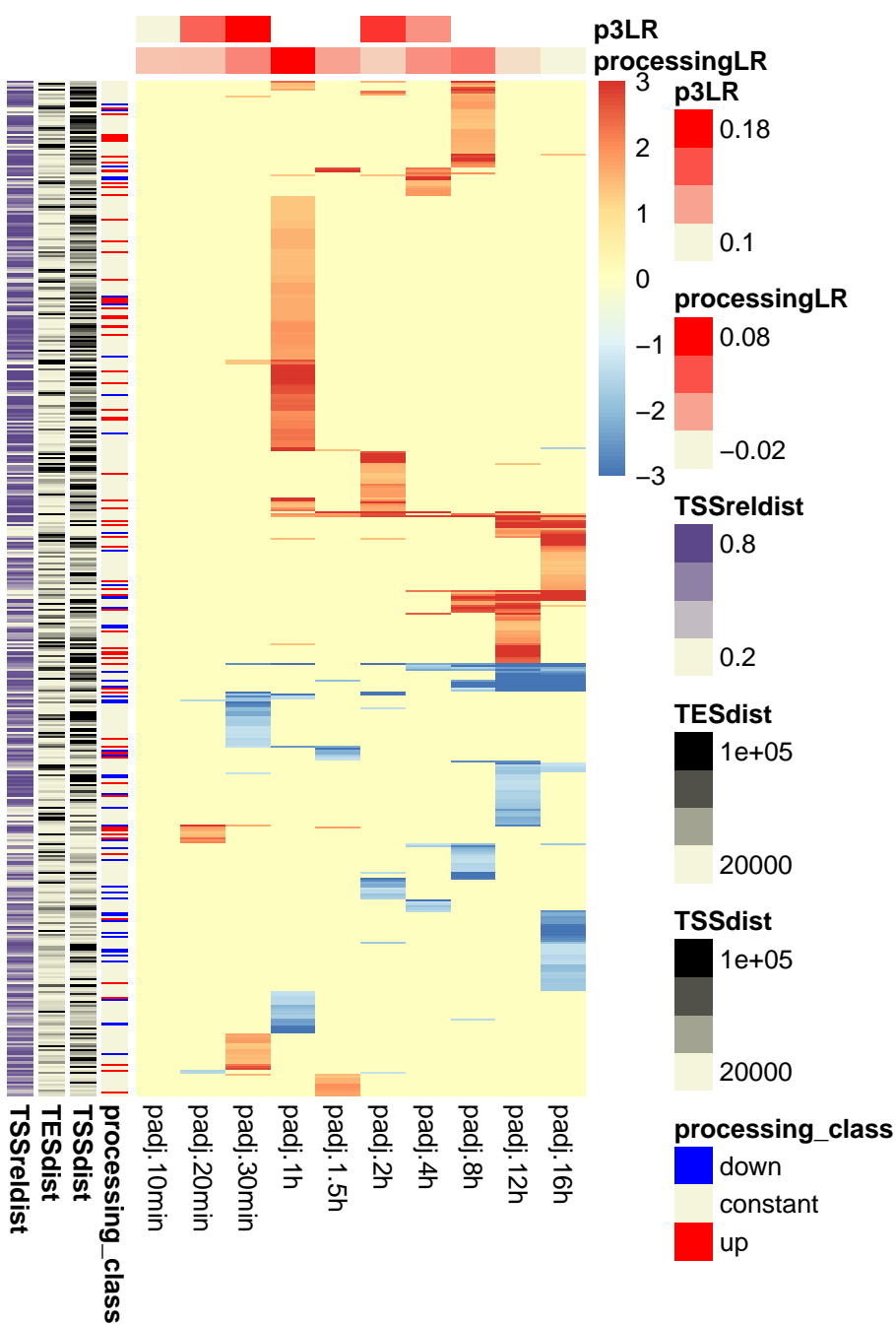

Supplement: Supplemental Material [file supp_gr.226035.117_Supplemental_Code.zip › dePretis2017_GR_code/figures/S4Gb.pdf]

# 216831 -

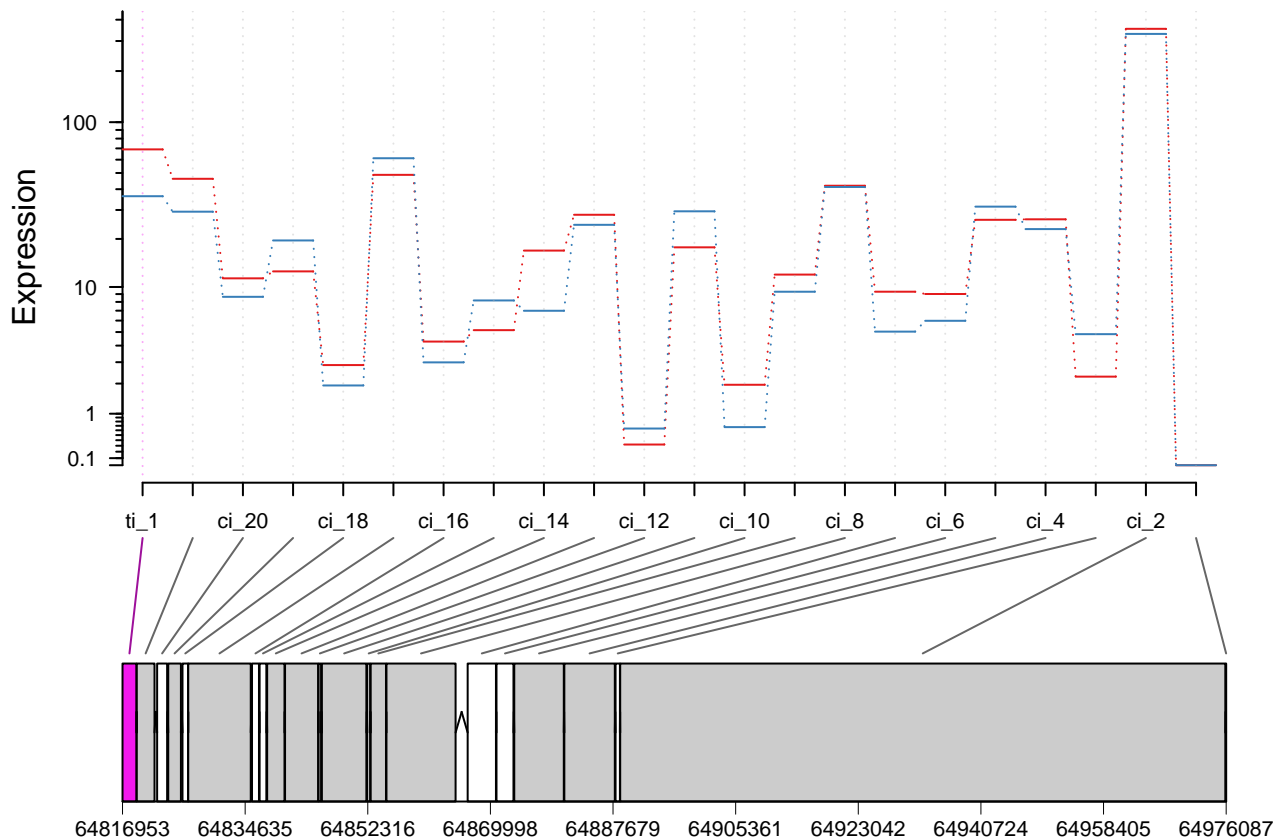

Supplement: Supplemental Material [file supp_gr.226035.117_Supplemental_Code.zip › dePretis2017_GR_code/figures/S4H.pdf]

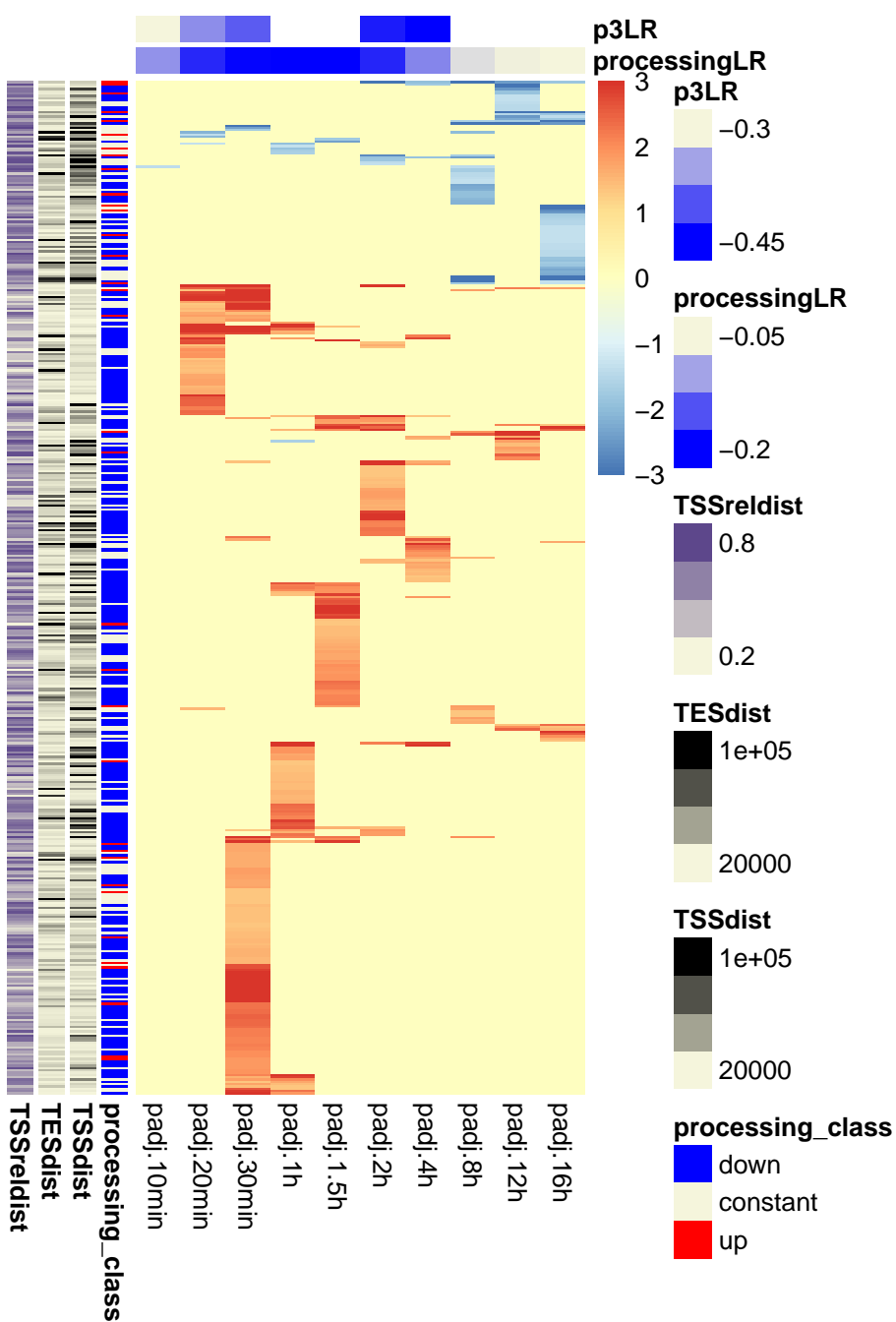

Supplement: Supplemental Material [file supp_gr.226035.117_Supplemental_Code.zip › dePretis2017_GR_code/figures/S4Ia.pdf]

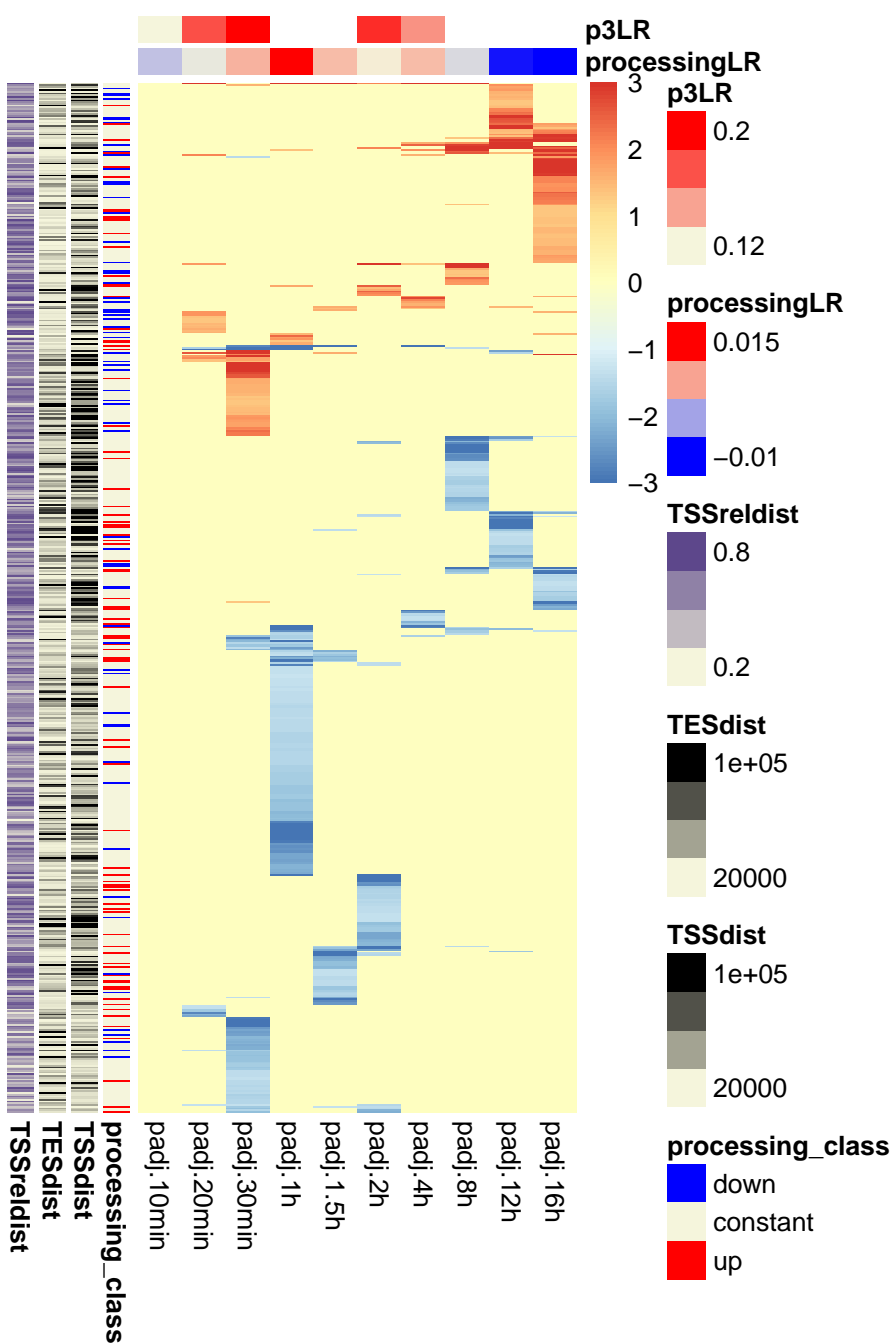

Supplement: Supplemental Material [file supp_gr.226035.117_Supplemental_Code.zip › dePretis2017_GR_code/figures/S4Ib.pdf]

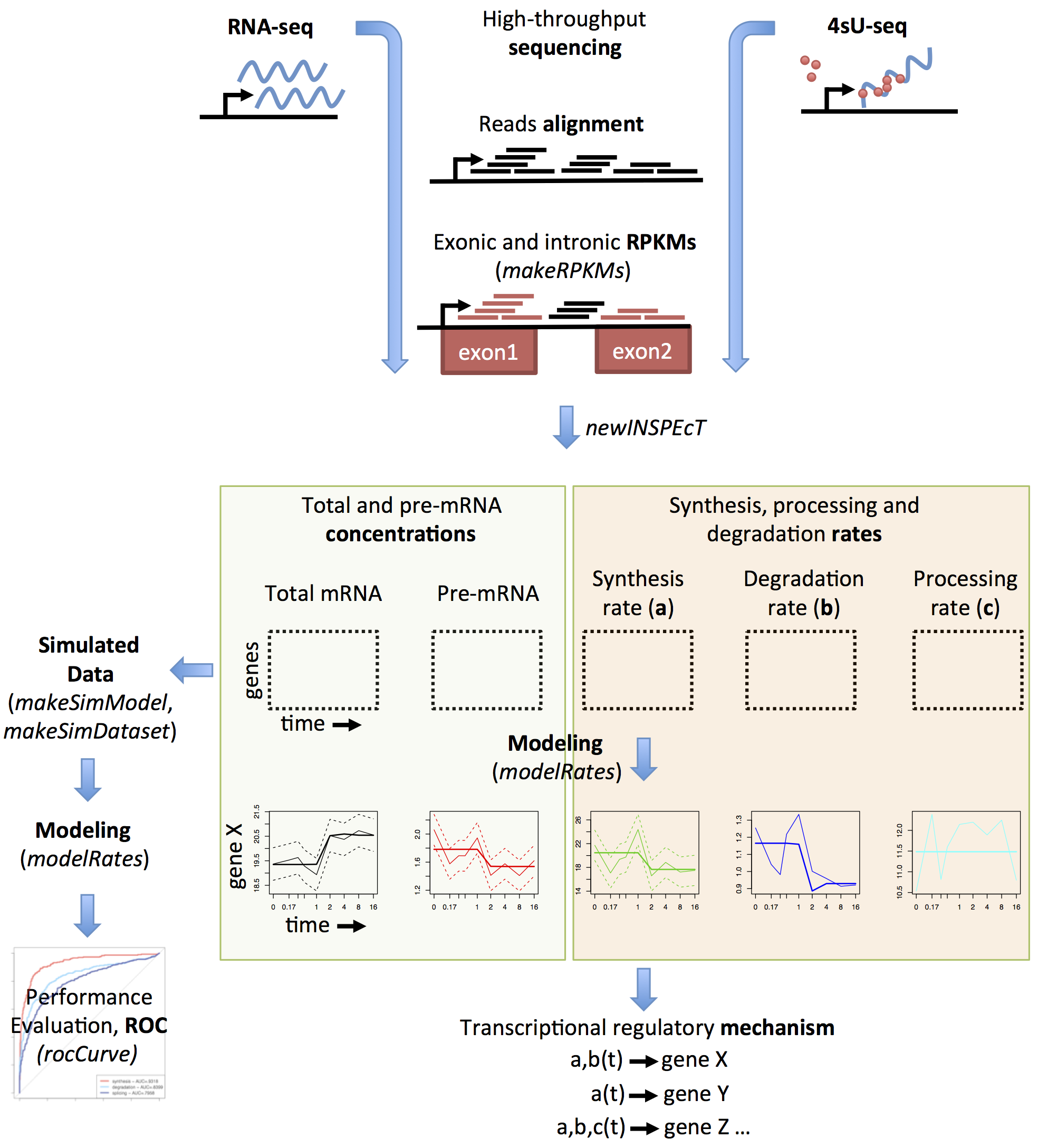

Supplement: Supplemental Material [file supp_gr.226035.117_Supplemental_Code.zip › dePretis2017_GR_code/primary_data_analysis/INSPEcT/vignettes/pipeline.png]
