# Supplementary material for: Integrative analysis of RNA polymerase II and transcriptional dynamics upon MYC activation
Source: Genome Res. 2017 Oct;27(10):1658–64. doi: 10.1101/gr.226035.117 (PMC5630029; doi:10.1101/gr.226035.117)
Supplement: Supplemental Material [file supp_gr.226035.117_Supplemental_Figures.docx]

**Figure S1.** Relationship between MYC binding and gene regulation. (*A*) For various systems, and for the specific comparisons indicated on the left side, the figure displays scatter plots of the variation of MYC ChIP-seq signal within promoters (x-axis, sampled within 25 uniformly sized bins), vs. the corresponding transcriptional variation (total RNA-seq, y-axis). Left and right columns are specific for repressed and induced genes, respectively. Linear models, Spearman correlations and the corresponding p-value are indicated for each plot. (*B*) For MYC dependent (left) and MYC independent genes (right) in the tet-MYC model, the figure displays density scatter plots (darker colors for higher density) of the variation of MYC ChIP-seq signal within promoters (x-axis), vs. the corresponding transcriptional variation (total RNA-seq, y-axis). The red, blue and orange lines capture the trend for induced, repressed, and both (combined) set of genes, respectively. The trend lines are based on local polynomial regression fitting, by excluding data points below the 2^nd^ and above the 98^th^ percentile of MYC binding variation. The title reports for the same set of genes the Spearman correlation. The vertical dashed lines identify the amount of MYC change at which promoters begin increasing their share of MYC binding (see panel *B*). Triangles mark outliers data points (top 0.5% of the data on both the x- and y-axis) that were forced within the plot range limits - the original values for these data points were used to derive the trend lines and correlation values). (*C*) Receiver Operating Characteristic (ROC) curves for the ability of discriminating induced and repressed genes at growing thresholds of changes in MYC binding, based on the genes in (*B*). AUC: area under the curve. The dots correspond to the variation of MYC at which promoters begin increasing their share of MYC binding. These identified the best trade-off between sensitivity and specificity, thus maximizing the ability to simultaneously classify induced and repressed genes. (*D*) For various systems, and for the specific comparisons indicated in the title, the figure displays density scatter plots (darker colors for higher density) of the log_2_ variation of at MYC promoter peaks (x-axis), vs the corresponding intensity in the baseline condition (y-axis). The trend lines are based on local polynomial regression fitting, by excluding data points below the 2^nd^ and above the 98^th^ percentile of MYC binding variation. The vertical solid lines indicate the point of no variation in MYC binding (i.e. saturation), while the vertical dashed lines indicate the level of MYC gain corresponding to an increase in the MYC share. Triangles mark outliers data points (top 0.5% of the data on both the x- and y-axis) that were forced within the plot range limits. (*E*) Receiver Operating Characteristic (ROC) curves for the ability of discriminating induced and repressed genes at growing thresholds of: (i) changes in the MYC share compared to control conditions (ΔMYCshare), (ii) ZBTB17 binding, (iii) a linear model combining Δ MYCshare and ZBTB17 binding, (iv) a linear model combining ΔMYCshare and MYC/ZBTB17 ratio. Apart from ΔMYCshare, MYC and ZBTB17 binding were quantified in the control conditions. Genes that were differentially expressed and bound by either MYC or ZBTB17 in the control or MYC-overexpression condition were considered (3400, 4215, and 3109 genes for U2OS^tet-MYC^, 3T9^MYC-ER^, and Eµ-*myc* models, respectively). AUC: area under the curve. (*F*) For each condition as in (a) scatter plot of the changes in the MYC share compared to control conditions (y-axis), compared with the ZBTB17 binding in the control condition (x-axis). Transcriptional induction and repression are color coded in red and blue, respectively, the darker the color the greater the modulation. The dashed black line depicts the ability of the MYC share to partition induced from repressed genes. The solid black line displays the linear model discriminating induced from repressed genes, based on the combined information from x- and y-axis, and the correlation between the linear model and transcriptional modulation is reported. (*G*) The differential expression in liver tumors induced by wild-type MYC (WT) and by a mutant defective in ZBTB17 binding (VD) is compared and displayed as histogram: primary (MYC-dependent) repressed genes have lower repression in MYC VD and primary induced genes have lower activation (Spearman p-values < 2.2e-16 in both for the average of the distributions being different from 0); all primary and secondary (MYC-independent) MYC responders are reported as background.

**Figure S2.** Time-course of MYC activation in 3T9^MYC-ER^ cells. (*A*) Western blots (representative of two independent experiments) showing the cellular localization of the MYC-ER protein before or after OHT addition for the indicated times. Vinculin was used as cytoplasmic marker protein, histone H3 as marker for the chromatin-bound fraction. The soluble fraction includes cytoplasm and nucleoplasm. (*B*) RT-PCR of mature vs. immature mRNA expression levels after MYC activation for the indicated time-points of the early OHT response. Primer location within exons (“E”) or introns (“I”) is indicated below the bars. Data are normalized to Rplp0 and plotted relative to the expression levels before OHT activation. (*C*) As in (*B*) for the late OHT response in the indicated genes and regions.

**Figure S3.** Dynamics of RNAPII loading and progression along transcriptional units: model evaluation. (*A*) The quality of partitioning the dataset in N clusters (2<N<40, x-axis) using k-means was evaluated. For each N, the quality of the clustering was determined using the Akaike information criterion (AIC, y-axis). The lower the AIC, the higher the quality of clustering (in terms of having high within-cluster homogeneity and low between-clusters similarity). The dashed line and arrow identify in 14 the optimal number of clusters, as further increasing N is not fully justified by a significant increase in cluster quality, see methods. (*B*) Left column: RNAPII flux at promoters (p_1_, solid lines for cl1-12); for each cluster, a black dashed line identifies flux levels of the untreated condition, while a grey dashed line at 0 separates positive (recruitment) from negative (loss) flux levels. Middle column: as the left column with restricted y-axis. Right column: modulation of the model rates p_2-4_ as log_2_ratio with respect to the untreated condition. (*C*) For each cluster and time point of OHT treatment, average density of RNAPII ChIP-seq reads (dots) within various portions of the transcriptional units, and the average synthesis rate. Solid lines represent the fit of the model. (*D*) Variance explained by the model for each cluster in a set of 10 fold cross-validations. (*E*) As in Supplementary Fig. 2a, Western blots (representative of two independent experiments) showing the cellular localization of the RNAPII before or after OHT addition for the indicated times.

**Figure S4.** Dynamics of RNAPII elongation and pre-mRNA processing: supplemental results. (*A*) For each intron (rows), the signal of 4sU-seq (left column) and RNAPII ChIP-seq (right column) normalized by the untreated condition is displayed for three particularly long induced and two repressed genes. Solid black lines highlight the trend by connecting at each time point the 1^st^ intron from the 5’ having a log_2_ratio lower than 0.4 (for the induced genes) and greater then -0.2 (for the repressed genes). (*B*) The dynamics of RNAPII in cluster 3 were re-assed after computationally imposing a constant elongation rate, set at the level of the untreated condition. The consequent fit of the model (solid line) to the data (dots) is displayed, including the average RNAPII ChIP-seq intensity in promoter, gene-body and TES, and average synthesis rate. Dashed lines indicate the fit of the model without constrained in the elongation rate. (*C*) Scatter plot of the time required reaching half of the maximum response for synthesis and processing rates. (*D*) Scatter plot of the half-response time for the pre-mRNA (x-axis) vs. the half-response time updated after imposing constant processing rates (y-axis). (*E*) Abundance of total and pre-mRNA, and the corresponding kinetic rates for *Slc16a1* (upper row); the ticker slid line indicate the fit of the models, and in the bottom row the models are updated after imposing a constant processing rate. (*F*) An example of reduced exon inclusion for an induced gene. (*G*) Heatmap of the events of altered exon inclusion (rows) following MYC-ER activation (columns) for induced (364 genes, left) and repressed genes (452, right). Events of increased and reduced exon inclusion are displayed on a red and blue color scale, respectively. For each event in each time point, the darker is the color the higher the significance of the alteration. The colorbar on the side of each heatmap display the position of the alteration in the CDS. (*H*) An example of increased intron retention for an induced gene. (*I*) Heatmap of the events of altered intron retention (rows) following MYC-ER activation (columns) for induced (385 genes, left) and repressed genes (522, right). Events of increased and reduced intron retention are displayed on a red and blue color scale, respectively. For each event in each time point, the darker is the color the higher the significance of the alteration. The colorbar on the side of each heatmap display the position of the alteration in the CDS.
